# *Akkermansia Muciniphila* Alleviates Severe Acute Pancreatitis via Amuc1409‐Ube2k‐Foxp3 Axis in Regulatory T Cells

**DOI:** 10.1002/advs.202504214

**Published:** 2025-06-04

**Authors:** Jinyan Xie, Lijun Du, Yunkun Lu, Xiuliu Guo, Xinyuan Zhou, Yifan Tong, Bo Shen, Xin Yu, Feng Guo, Hong Yu

**Affiliations:** ^1^ Department of Critical Care Medicine Sir Run Run Shaw Hospital Zhejiang University School of Medicine Hangzhou 310016 P. R. China; ^2^ Zhejiang Key Laboratory of Precise Diagnosis and Treatment of Abdominal Infection Sir Run Run Shaw Hospital School of Medicine Zhejiang University Hangzhou 310016 P. R. China; ^3^ Department of General Surgery Sir Run Run Shaw Hospital Zhejiang University School of Medicine Hangzhou 310016 P. R. China; ^4^ Department of Anesthesiology Sir Run Run Shaw Hospital Zhejiang University School of Medicine Hangzhou 310016 P. R. China

**Keywords:** akkermansia muciniphila, Amuc_1409, regulatory T cells, severe acute pancreatitis, Ube2k

## Abstract

Severe acute pancreatitis (SAP) is a critical condition characterized by an imbalance between systemic inflammatory response syndrome (SIRS) and compensatory anti‐inflammatory response syndrome (CARS). Disruptions in intestinal microbiota exacerbate this imbalance; however, the mechanisms by which commensal bacteria regulate SAP‐induced SIRS and/or CARS remain unclear. This study reports that the abundance of the gut commensal *Akkermansia* is significantly reduced in fecal samples from patients with acute pancreatitis (AP) and is inversely associated with the severity of systemic inflammatory responses. Using Foxp3‐DTR and IL‐10‐KO mice, it is found that *Akkermansia muciniphila* and its derived protein Amuc_1409 can suppress SAP‐induced pancreatic and systemic inflammation by increasing peripheral regulatory T cells (Tregs) and enhancing the IL‐10 expression. Mechanistically, Amuc_1409 interacts with Ube2k to reduce the ubiquitination degradation of Foxp3, and it promotes the differentiation of Tregs and the production of IL‐10, effectively alleviating inflammation induced by SAP. These findings highlight *A. muciniphila* and its derivative Amuc_1409 as promising probiotic and biomolecule for managing acute pancreatitis and potentially other inflammatory conditions.

## Graphical abstract

*Akkermansia* depletion correlates with acute pancreatitis (AP) severity. *A. muciniphila* and its protein Amuc_1409 mitigate pancreatic/systemic inflammation by expanding Tregs and IL‐10. Mechanistically, Amuc_1409 binds Ube2k, inhibiting Foxp3 ubiquitination to stabilize Treg differentiation and IL‐10 production, countering SAP‐induced SIRS/CARS imbalance. These findings highlight  *A. muciniphila* and Amuc_1409 as therapeutic candidates for AP and inflammatory disorders.

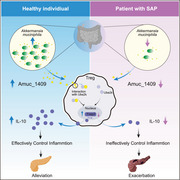

## 1 Introduction

AP is one of the most common gastrointestinal diseases, and its incidence rate has been increasing.^[^ ^1^ ^]^ AP can be classified into mild acute pancreatitis (MAP), mild severe acute pancreatitis (MSAP), and SAP based on the extent of inflammation. MAP is primarily characterized by interstitial pancreatic edema and rarely results in death. However, 15–25% of patients with MAP may progress to SAP, which is marked by severe systemic inflammatory responses and persistent multiple organ failure (PMOF). Due to the complex etiology, the lack of specific clinical drugs, and the absence of effective intervention, SAP has a poor prognosis, with a mortality rate as high as 36–50%.^[^ ^2^ ^]^

Cumulative evidence strongly supports that premature activation of trypsinogen is a critical pathological mechanism leading to acinar cell necrosis.^[^ ^3^ ^]^ Damaged acinar cells release chemokines and cytokines, which recruit and mediate the infiltration of monocytes and neutrophils to the injury site.^[^ ^4^ ^]^ Subsequently, monocytes were activated by damage‐associated molecular patterns (DAMPs) released from necrotic acinar cells, leading to an amplified production of pro‐inflammatory cytokines. Macrophages in distant organs are also activated, exacerbating systemic inflammation and causing distant organ injury, which contributes to the development of SIRS and PMOF.^[^ ^5^ ^]^ This phase of hyperinflammation is often followed by a CARS mediated by an increase in Tregs, aimed at resolving the deadly cytokine storm, allowing most patients to restore a balance between hyperinflammatory response and immunosuppression.^[^ ^6^ ^]^ However, if this homeostasis is disrupted, the patient may succumb to either an early, overwhelming inflammatory response or to late‐stage immunosuppression.^[^ ^7^ ^]^ Currently, there remains no effective strategy to control the excessive inflammatory response or to reduce mortality in SAP.

SAP is often accompanied by increased intestinal permeability and mucosal barrier damage, resulting in bacterial translocation and even abdominal infection.^[^ ^8^ ^]^ Our previous study indicated that perturbation of the intestinal microbiota was a critical factor to exacerbate pancreatic and systemic inflammatory responses.^[^ ^9^ ^]^ Furthermore, there is growing evidence that metabolites and proteins of commensal bacteria attenuate SAP‐induce SIRS by suppressing the inflammatory mediated signaling of macrophages and neutrophils.^[^ ^9^, ^10^ ^]^ Unfortunately, our understanding of how commensal bacteria regulate SAP‐induced CARS is limited. Additionally, the upstream mechanisms and downstream targets through which commensal bacteria influence the function of Tregs in AP remain poorly understood.

Here, we screened the intestinal microbiome of patients with AP and identified that the abundance of *Akkermansia* genus was significantly reduced in patients with AP than in healthy control. Subsequently, we discovered that *Akkermansia muciniphila* and its derivative Amuc_1409 could interact with Ube2k to induce Foxp3 expression in Tregs, which produces anti‐inflammatory cytokine, IL‐10, to ameliorate SAP severity by suppressing pancreatic and systemic inflammatory responses.

## 2 Results

### 2.1 Patients with AP Exhibit a Decreased Fecal Abundance of *Akkermansia*

**Figure 1:**
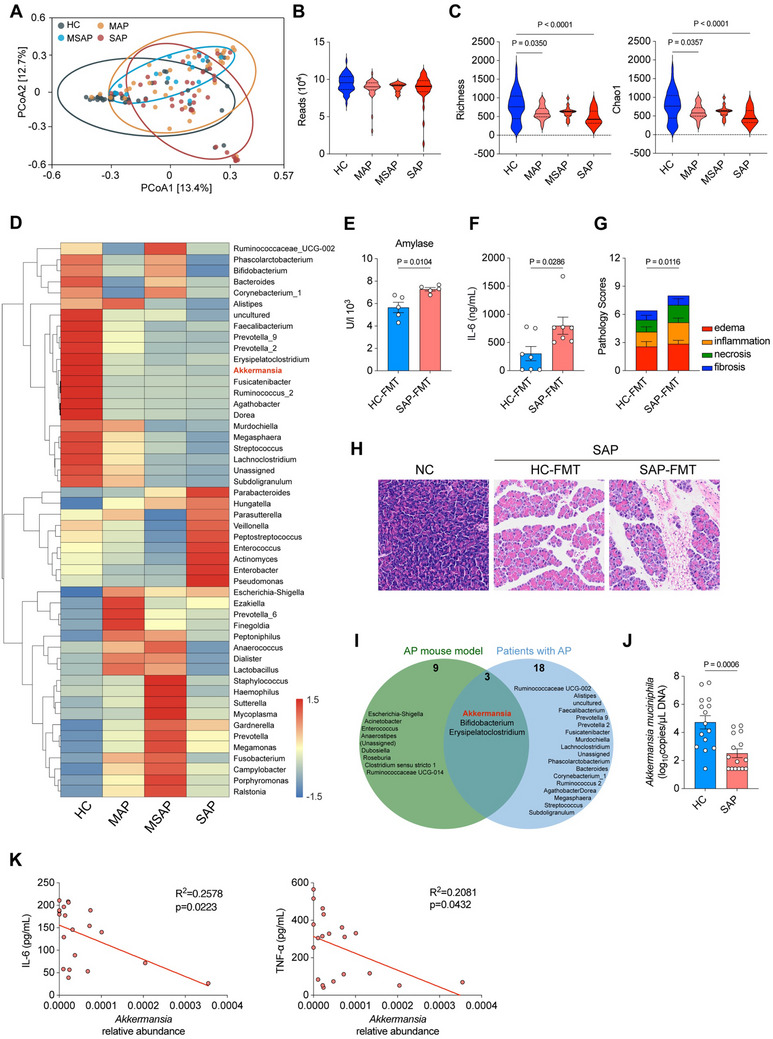
Patients with AP exhibit a decreased fecal abundance of *Akkermansia* that was inversely correlated with the inflammation of AP. The 16S rRNA sequencing analysis of fecal samples from patients with AP or Healthy control. A) PCoA, B) total reads, C) Alpha diversity indicators of Richness (left) and Chao (right) among the four groups (HC *n* = 46, MAP *n* = 38, MSAP *n* = 16, SAP *n* = 32). D) The heatmap of relative abundance of bacteria at the genus level. E) The concentration of serum amylase (*n* = 5), F) serum IL‐6 level (*n* = 7), G) pathology scores, and H) pancreatic histopathology from Abx‐treated mice received FMT from HC or patients with SAP donors at 12 h post caerulein‐induced SAP modeling. I) Venn diagram analysis between the gut bacteria reduced in AP mouse models and those in AP patients. J) Genomic copies of *A. muciniphila* (*n* = 15). K) The correlations between the concentrations of IL‐6 (left) and TNF‐α (right) and the relative abundance of *Akkermansia*. The two‐sided *p‐*values were examined by one‐way ANOVA with Dunnett's multiple comparisons test (C) or Student's *t*‐test (E–G, J) and data were presented as mean ± sem. *R^2^* and exact two‐sided *p‐*values calculated by Pearson's test are shown (K).

Previous studies have identified patients with AP exhibit intestinal flora dysregulation, but the role of intestinal flora in the progression of AP is unknown. Here, we collected fecal samples from 86 hospitalized patients (38 with MAP, 16 with MSAP, and 32 with SAP) and 46 healthy control volunteers (HC). There was no significant difference in age, sex, lifestyle, etiology or antibiotics use of AP between AP groups (**Table** **1**) as determined by logistic regression. Patients with MAP (31.6%) and MSAP (37.5%) had a higher recurrence rate, compared with those with SAP (6.3%) (Table 1). Then, we analyzed the difference of their fecal microbiota composition by 16S rRNA sequencing. Principal coordinate analysis (PCoA) of all samples revealed a different microbial signature of patients with AP compared with HC, with the most prominent changes showed between patients with SAP and HC (**Figure** **1**A). There were no significant differences in reads between the groups, but a remarked reduced richness and Chao1 was observed in patients with MAP and SAP compared with HC (Figure 1B,C). The heatmap exhibited a reduced abundance of *Akkermansia*, *Lachnoclostridium*, and *Ruminococcus*, and an increasing abundance of *Enterococcus*, *Pseudomonas*, and *Escherichia‐Shigella* in the patients with AP compared with the HC (Figure 1D). To validate the critical role of gut microbiota in mediating the progression of AP, we performed fecal microbiota transplantation (FMT) experiment. Specifically, fecal samples from SAP patients and healthy individuals were orally administered to antibiotic‐treated (Abx) mice. After 48 h of microbial colonization, a murine SAP model was established using caerulein. Compared to mice receiving FMT from healthy donors, the SAP patient FMT group exhibited significantly elevated serum amylase levels, increased expression of inflammatory factors (*Il6, Il1b*) in both pancreatic tissue and serum, and markedly aggravated pancreatic pathological damage, including acinar cell edema, necrosis, and inflammatory infiltration (Figure 1E—H; Figure S1A, Supporting Information). These findings suggest that gut microbiota alterations in AP patients are a key factor mediating disease severity.

**Table 1:** Clinical characteristics of patient with acute pancreatitis.

Property	Level of pancreatitis			*p*‐value^*^ ^)^
	Mild (n = 38)	Moderately severe (n = 16)	Severe (n = 32)	
Age, y, mean (range)	47±14.9 (20‐78)	50±12.6 (25‐82)	50±16.9 (20‐88)	0.65
Sex, f/m	9/29	4/12	11/21	0.6023
Etiology, n (%)				
Gallstone	18 (47.4)	5 (31.3)	21 (65.6)	0.0734
Alcohol	1 (2.6)	1 (6.3)	1 (3.1)	0.7802
hyperlipemia	17 (44.7)	9 (56.3)	11 (34.4)	0.3602
Others^#^ ^)^	2 (5.3)	1 (6.3)	0 (0)	0.4059
Lifestyle, n (%)				
Smoking	12 (31.6)	3 (18.8)	3 (9.4)	0.0760
Drinking	14 (36.8)	5 (31.3)	7 (21.9)	0.3557
IV antibiotics on admission	2 (5.3)	1 (6.3)	1 (3.1)	0.9999
Previous pancreatitis	12 (31.6)	6 (37.5)	2 (6.3)	0.0097

*) *p*‐value calculated using Fisher's exact probability test between mild, moderately severe, and severe groups.

^#)^ Other = Idiopathic, traumatic pancreatitis.

Subsequently, we conducted a joint analysis of 16S rRNA data from previous AP mouse models.^[^ ^9^ ^]^ By performing Venn diagram analysis between the gut bacteria reduced in AP mouse models and those in AP patients, we identified three co‐altered bacterial species: *Akkermansia*, *Bifidobacterium*, and *Erysipelatoclostridium* (Figure 1I). Notably, the genomic copies and relative abundance of *Akkermansia muciniphila* (*A. muciniphila*) was significantly decreased in the SAP group compared with HC (Figure 1J; Figure S1B, Supporting Information). Then, we determined the expression of inflammatory cytokines, IL‐6 and TNF‐α in the serum of patients with AP, and correlation analysis further revealed that the relative abundance of *A. muciniphila* in fecal samples showed negative correlations with pro‐inflammatory cytokines (IL‐6 and TNF‐α) (Figure 1K). Furthermore, the serum concentrations of C‐reactive protein (CRP) and amylase inversely correlated with the abundance of *Akkermansia* in patients with AP (Figure S1C,D, Supporting Information). Taken together, these data suggested that *Akkermansia* was inversely correlated with the inflammation of AP.

### 2.2 *Akkermansia Muciniphila* Protects Against SAP by Attenuating Pancreatic and Systemic Inflammation

**Figure 2:**
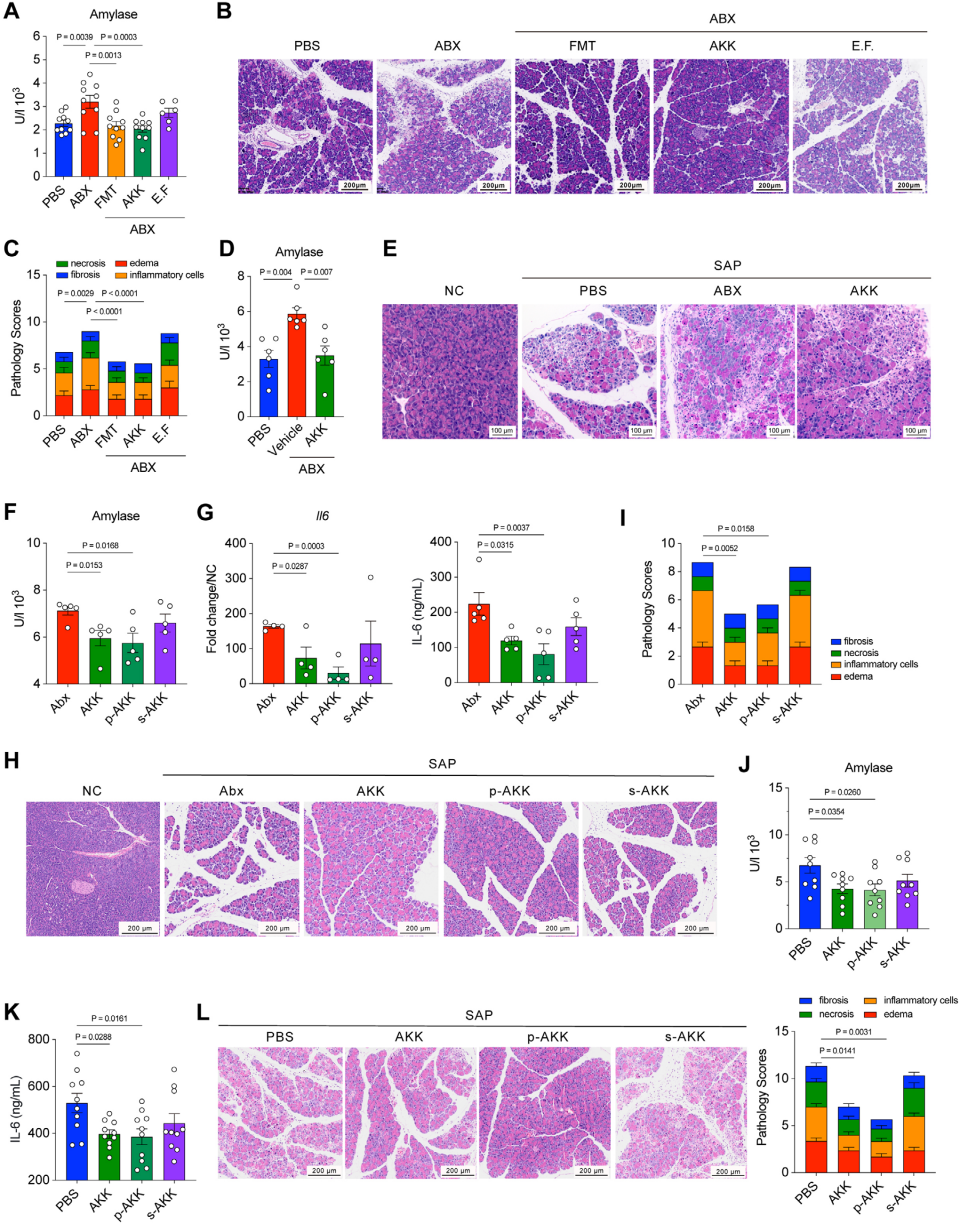
*Akkermansia muciniphila* protects the host from MAP and SAP by attenuating pancreatic and systemic inflammation. A) The concentration of serum amylase, B) pancreatic histopathology, and C) pathology scores from mice treated with PBS, Abx, or Abx‐treated mice received FMT or colonized with *Akkermansia muciniphila* (AKK) or E.F at 12 h post caerulein‐induced SAP modeling (*n* = 6–10). D) The serum amylase, E) pancreatic histopathology from mice treated with PBS, Abx, or Abx‐treated mice colonized with AKK at 3 days post L‐arginine‐induced SAP modeling (*n* = 6). F) Serum amylase level (*n* = 5), G) pancreatic Il6 mRNA level (*n* = 4) and serum IL‐6 level (*n* = 5), H) pancreatic histopathology, and I) pathology scores from mice treated with Abx or Abx‐mice treated with AKK, pasteurized *A. muciniphila* (p‐AKK), supernatant *A. muciniphila* (s‐AKK) at 12 h post caerulein‐induced SAP modeling. J) Serum amylase level (*n* = 9), K) serum IL‐6 level (*n* = 10), L) pancreatic histopathology, and pathology scores from L‐arginine‐induced SAP mice treated with AKK, p‐AKK, s‐AKK at 3 days post modeling. The two‐sided *p‐*values were examined by one‐way ANOVA with Dunnett's multiple comparisons test and data were presented as mean ± sem (A, C,D, F,G, I–L).

Our previous work also has identified *Akkermansia* could be a potential probiotic that alleviates AP in mice.^[^ ^9^ ^]^ To test the potential probiotic role of *Akkermansia* during the progression of AP, we used highly concentrated broad‐spectrum antibiotic (Abx) by oral gavage to deplete commensal bacteria and constructed AP mouse model by caerulein injection (Figure S2A, Supporting Information). As previously reported,^[^ ^9^ ^]^ Abx‐treated mice displayed a distinct microbial profile, as revealed by PCoA, with significantly diminished α‐diversity indices (Richness and Chao1) when compared to PBS‐treated controls (Figure S2E,F, Supporting Information). Notably, Abx‐treated mice demonstrated substantial alterations in microbial composition, characterized by decreased relative abundances of beneficial taxa including *Akkermansia*, *Lactobacillus*, and *Ruminococcaceae*, concomitant with an enrichment of potentially pathogenic genera such as *Pseudomonas*, *Sphingomonas*, and *Escherichia‐Shigella* (Figure S2G,H, Supporting Information). Furthermore, Abx‐treated mice exhibited a significantly higher content of serum amylase and more severe pancreatic inflammatory lesions than PBS‐treated controls in SAP models. FMT from PBS‐treated mice into Abx‐treated mice reversed the amylase content and improved pancreatic pathological lesions (**Figure** **2**A–C), suggesting that intestinal microbiota alteration influenced the disease progression. Then, we colonized *A. muciniphila* or *Enterococcus faecalis* (E.F) to Abx‐treated mice and found that *A. muciniphila* colonization, not E.F colonization, markedly reduced serum amylase concentration and alleviated pancreatic edema and inflammatory cells infiltration (Figure 2A–C). Due to the complex pathogenic factors of AP, multiple animal model validations are required. We then used L‐arginine to construct another SAP mouse model, as described previously^[^ ^11^ ^]^ (Figure S2B, Supporting Information). *A. muciniphila* colonization significantly reduced serum amylase level and improved pancreatic pathological lesion (Figure 2D,E). The two different mouse models suggested the preventive role of *A. muciniphila* on SAP.

To determine the protective component of *A. muciniphila*, we filtered the supernatant of *A. muciniphila* (s‐AKK, comprised by metabolites) and pasteurized component of *A. muciniphila* (p‐AKK, comprised by proteins), and gavaged to Abx‐treated mice (Figure S2A, Supporting Information). Intriguingly, the treatment of p‐AKK significantly alleviated SAP‐induced serum amylase and downregulated IL‐6 expression in the local pancreas and systemic circulation (Figure 2F,G). Notably, p‐AKK administration significantly attenuated pancreatic edema and inflammatory cell infiltration. In contrast, while oral s‐AKK treatment demonstrated a modest reduction in SAP‐induced inflammatory response and pancreatic tissue damage, these protective effects failed to achieve statistical significance (Figure 2H,I). This differential efficacy strongly suggests that the observed benefits were primarily mediated by microbial proteins rather than bacterial metabolites. To further explore the therapeutic effect of *A. muciniphila*, we constructed SAP mouse model using L‐arginine, and gavaged with *A. muciniphila*, p‐AKK or s‐AKK daily until the end of the experiments (Figure S2B, Supporting Information). As previously observed, *A. muciniphila* and p‐AKK exhibited a significant reduction of serum amylase and IL‐6 expression (Figure 2J,K; Figure S2I, Supporting Information), and an observable improvement of pancreatic edema, necrosis, and inflammatory cell infiltration (Figure 2L). Together, these concordant results in two different SAP mouse models indicate a protective and therapeutic role of *A. muciniphila*‐driven proteins in alleviating SAP‐induced inflammatory responses and pathological lesion in the local pancreas and systemic circulation.

### 2.3 *Akkermansia Muciniphila* Upregulates Peripheral Regulatory T Cells and Promotes Intestinal Barrier Integrity

To explore the regulation of *A. muciniphila* on immune cells, we performed single‐cell RNA sequencing of peripheral blood mononuclear cells (PBMCs) from L‐arginine‐induced SAP mice, *A. muciniphila*‐treated SAP mice and non‐treated control to identify the key cells regulated by *A. muciniphila*. After strict quality control and filtering (Figure S3A, Supporting Information), we obtained 26 176 single cells from these samples. Five major cell clusters were identified, including B cells (*Ms4a1* ^+^), CD4^+^ T cells (*Cd4^+^*), CD8^+^ T cells (*Cd8a* ^+^), myeloid cells (*Cd14* ^+^), and natural killer (NK) cells (*Nkg7* ^+^) (Figure S3B, Supporting Information). Then, we analyzed the immune cell composition in each of the individuals and noted that the percentage of myeloid cells were significantly increased in SAP mice treated with or without *A. muciniphila*, compared with negative control (NC) mice (Figure S3C, Supporting Information). We subdivided myeloid cells into mononuclear macrophage (Mono‐mac), neutrophil, mast cells and proliferative cells (Figure S3D,E, Supporting Information), and noted that the percentage of mononuclear macrophages was decreased in the *A. muciniphila*‐treated SAP mice, compared with SAP mice (Figure S3F, Supporting Information). Previous studies have demonstrated that activated macrophages and neutrophils play a crucial role as key immune cells in initiating and amplifying the inflammatory response induced by SAP.^[^ ^5^, ^9^, ^10^ ^]^ To determine whether *A. muciniphila* alleviates the inflammatory response by inhibiting macrophages and neutrophils, we examined the changes of peripheral macrophages and neutrophils using flow cytometry. Expectedly, SAP significantly upregulated frequency of peripheral macrophages and neutrophils, but *A. muciniphila* group did not exhibit lower frequency of peripheral macrophages and neutrophils, compared with SAP group (Figure S4A,B, Supporting Information), suggesting that *A. muciniphila* may alleviate acute pancreatitis through the involvement of other immune cells.

**Figure 3:**
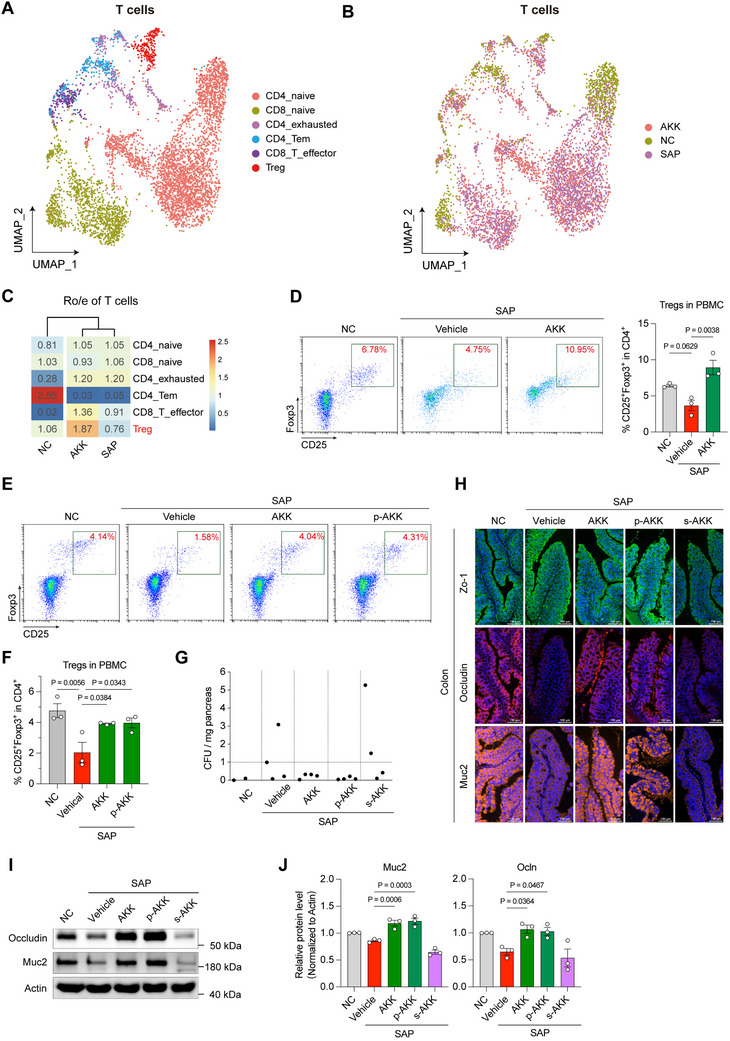
*Akkermansia muciniphila* upregulated peripheral regulatory T cells and promotes intestinal barrier integrity. L‐arginine‐induced SAP mice treated with AKK daily for 3 days. PBMCs were isolated at 3 days post modeling and single‐cell transcriptomics sequencing was performed. A) UMAP plots of T cells, including six major clusters. B) UMAP plots of T cells, C) Ro/e of T cells, D) the percentage of CD4^+^/CD25^+^/Foxp3^+^‐Tregs in PBMC from NC mice, SAP mice and AKK‐treated SAP mice (*n* = 3). Mice treated with Abx or Abx‐treated mice colonized with AKK or p‐AKK at 12 h post caerulein‐induced SAP modeling. E) Representative flow cytometry plots, F) the percentage of CD4^+^/CD25^+^/Foxp3^+^‐Tregs (*n* = 3). L‐arginine‐induced SAP mice treated with AKK, p‐AKK or s‐AKK daily for 3 days. G) Colony forming units (CFU) were counted from pancreas homogenates and exceeded the cut‐off level of 1 CFU/mg tissue weight (*n* = 2–4). H) Representative indirect fluorescent assay (IFA) images of colon. Zo‐1, Occludin, and Muc2 as makers for tight junction proteins. I) Representative Western blot images of Occludin, Muc2 and Actin in colon. J) Quantification of the proteins in Figure 3I. The protein levels of Muc2 (left) and Ocln (right) were normalized to those of actin. The amount of each protein in the NC group was defined as 1 (*n* = 3). The two‐sided *p‐*values were examined by one‐way ANOVA with Dunnett's multiple comparisons test and data were presented as mean ± sem (D, F, J).

It's worth noting that the percentage of CD4^+^ T cells and CD8^+^ T cells was decreased in *A. muciniphila*‐treated SAP mice, compared with SAP mice (Figure S3C, Supporting Information). Subsequently, we subdivided T cells into CD4 naïve cells, CD8 naïve cells, CD4 exhausted cells, CD4 Tem cells, CD8 T effector cells, and Tregs (**Figure** **3**A; Figure S3G, Supporting Information). Then, we analyzed the changes of T cell subtypes in each of groups and noted that the frequency of CD4 exhausted cells and CD8 T effector cells was enriched and the percentage of CD4 Tem cells were decreased in SAP mice, compared with NC mice. Interestingly, we found that the percentage of Tregs was decreased in the SAP mice and more enriched in the *A. muciniphila*‐treated SAP mice, compared with NC mice (Figure 3B,C), and confirmed this phenomenon by flow cytometry (Figure 3D), implying *A. muciniphila* may alleviate SAP‐induced inflammation via upregulating the percentage of peripheral Tregs. We further determined the immunomodulatory effects of *A. muciniphila* in another caerulein‐induced SAP model, and detected the changes of macrophages, neutrophils, and Tregs in PBMC and splenic cells by flow cytometry. As expected, the occurrence of SAP led to a substantial increase in the percentage of neutrophils and macrophages in PBMC (Figure S4C,D, Supporting Information) and splenic cells (Figure S5A,B, Supporting Information), and neither *A. muciniphila* nor p‐AKK reversed these changes. Notably, *A. muciniphila* and p‐AKK could restore the decreased Treg levels in PBMCs and splenic cells of SAP mice to normal levels (Figure 3E,F; Figure S5C, Supporting Information).

In addition to hyper‐inflammatory responses, SAP often accompanies intestinal dysfunction, which in severe cases can lead to intestinal failure and bacterial translocation.^[^ ^8^ ^]^ Hence, we determined the bacterial infection of pancreas by colony growth assays of the tissue homogenate on agar plates and detected a marked bacterial load in the pancreas of SAP mice, exceeding 1 colony‐forming unit (CFU) per milligram of pancreas. *A. muciniphila* and p‐AKK, not s‐AKK, were reduced bacterial loads in the pancreas (Figure 3G), suggesting that *A. muciniphila* and p‐AKK effectively reduce bacterial translocation. To further evaluate the effect of *A. muciniphila* on the intestinal barrier, we tested pathogenic lesion by H&E staining, and analyzed the expression of structural protein components of the intestinal barrier like tight junctions by immunofluorescence assay (IFA) and Western blot. The pathogenic lesion of duodenum and colon was observed in SAP mice and s‐AKK‐treated SAP mice, while it was alleviated in *A. muciniphila*‐ and p‐AKK‐treated SAP mice (Figure S5D, Supporting Information). Furthermore, we observed a significant reduction of mRNAs encoding for mucins 2 (*Muc2*), Occluding (*Ocln*) in the colon of SAP mice, whereas those mRNA levels were markedly upregulated after treating with *A. muciniphila* and p‐AKK (Figure S5E, Supporting Information). The protein expression of those tight junction proteins, Muc2 and Ocln, not Zo‐1, were also downregulated in the duodenum and colon of SAP mice and s‐AKK‐treated SAP mice, and restored in *A. muciniphila*‐ and p‐AKK‐treated SAP mice (Figure 3H—J; Figure S5F, Supporting Information), implying that *A. muciniphila* and its protein components promote intestinal barrier integrity. Together, those results indicated a role for *A. muciniphila*‐driven proteins in suppressing inflammatory responses via upregulating Tregs and preventing bacterial translocation via promoting intestinal barrier integrity.

### 2.4 *Akkermansia Muciniphila*‐Driven Protein Amuc_1409 Protects the Host from SAP by Dampening Pancreatic and Systemic Inflammation

**Figure 4:**
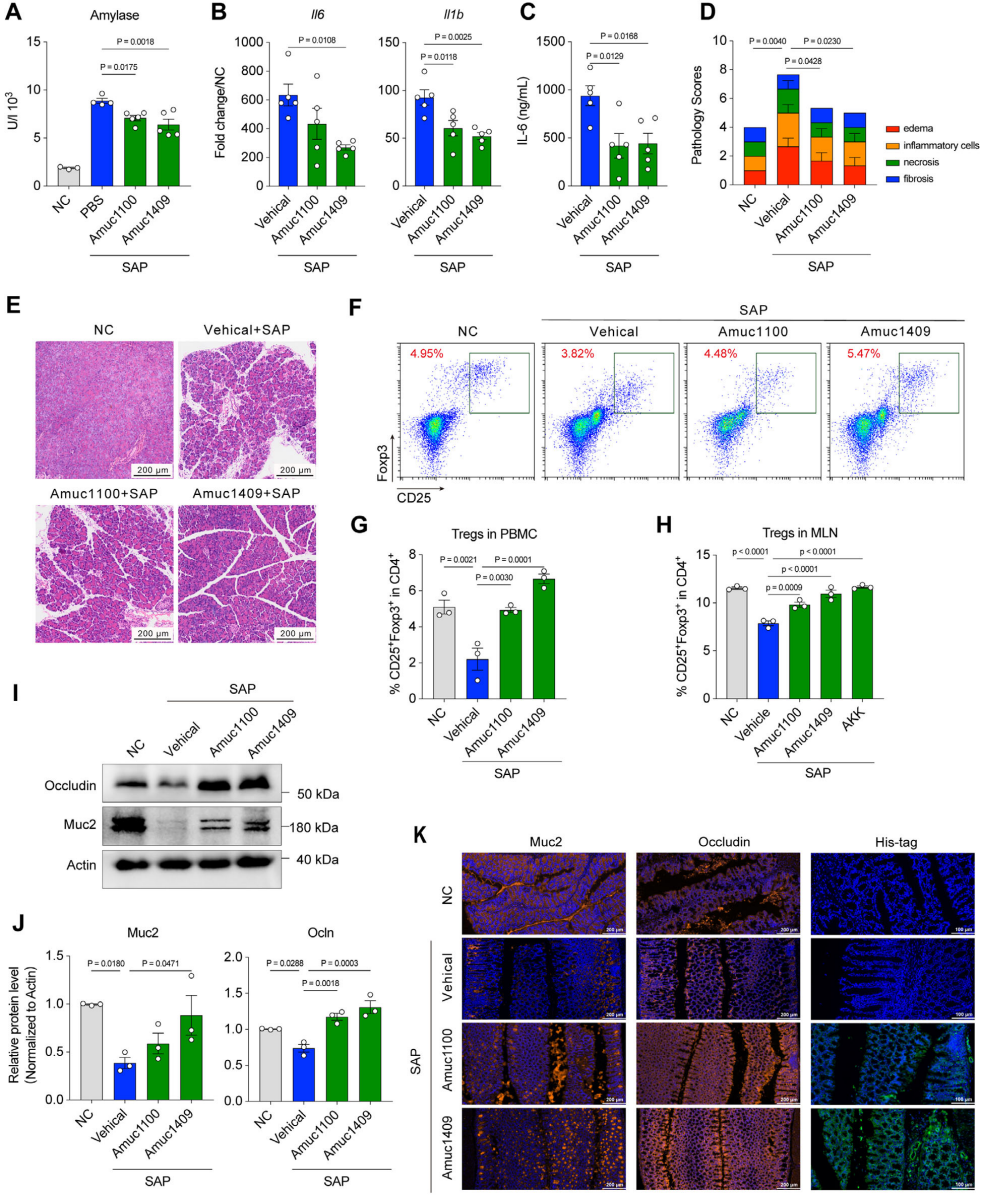
*A. muciniphila*‐driven protein Amuc_1409 protects the host from SAP by dampening pancreatic and systemic inflammation. Mice treated with Abx or Abx‐treated mice gavaged with Amuc_1100 or Amuc_1409 at 12 h post caerulein‐induced SAP modeling. A) Serum amylase level (*n* = 3–5). B) Pancreatic Il6, Il1b mRNA level (*n* = 5) and C) serum IL‐6 level (*n* = 5). D) Pathology scores (*n* = 3) and E) pancreatic histopathology. F) Representative flow cytometry plots, G) the percentage of CD4^+^/CD25^+^/Foxp3^+^‐Tregs in PBMC (*n* = 3). H) The percentage of CD4^+^/CD25^+^/Foxp3^+^‐Tregs in MLN in L‐arginine‐induced SAP model (*n* = 3). I) Representative Western blot images of Occludin, Muc2, and Actin in colon. J) Quantification of the proteins in Figure 4I. The protein levels of Muc2 (left) and Ocln (right) were normalized to those of Actin. The amount of each protein in the NC group was defined as 1 (*n* = 3). K) Representative IFA images of colon. Occludin, Muc2 as makers for tight junction proteins, His‐tagged for Amuc_1100 and Amuc_1409. The two‐sided *p‐*values were examined by one‐way ANOVA with Dunnett's multiple comparisons test and data were presented as mean ± sem (A–D, G,H, J).

Eun‐Jung Kang et al. performed comprehensive proteomic profiling of *A. muciniphila* secretory proteins using nanoflow liquid chromatography‐tandem mass spectrometry (nLC‐MS/MS). Their quantitative analysis revealed Amuc_1409 as the most abundant secreted protein, based on both MS/MS spectral counts and intensity‐based absolute quantification (iBAQ) values.^[^ ^12^ ^]^ Notably, Amuc_1100— a previously characterized multifunctional outer membrane protein of *A*. *muciniphila*—has been demonstrated to attenuate AP by reducing macrophage and neutrophil infiltration.^[^ ^10^, ^13^ ^]^ To test whether *A. muciniphila* has a protective effect on SAP mediated by Amuc_1409, we treated Abx mice with purified His‐tagged Amuc_1409 and Amuc_1100 produced in *Escherichia coli* (*E. coli*) and constructed SAP models using caerulein, in which Amuc_1100 treatment as positive control (Figure S2A, Supporting Information). Treated with Amuc_1409 or Amuc_1100 significantly reduced serum amylase concentration (**Figure** **4**A) and downregulated IL‐6 expression in the local pancreas and systemic circulation (Figure 4B,C). Moreover, Amuc_1409 or Amuc_1100 treatment remarkedly alleviated pancreatic edema and inflammatory cell infiltration (Figure 4D,E). Amuc_1409 also demonstrated significant protective effects in both caerulein‐induced MAP and L‐arginine‐induced SAP models (Figure S6A–G, Supporting Information). Treatment with Amuc_1409 or Amuc_1100 promoted peripheral Tregs proportion in caerulein‐induced (Figure 4F,G) and L‐arginine‐induced SAP model (Figure S6H,I, Supporting Information), and Amuc_1409 exhibited a stronger promotion on Tregs than Amuc_1100.

Mesenteric lymph nodes (mLNs) are highly efficient sites for the peripherally induced Tregs.^[^ ^14^ ^]^ Furthermore, commensal bacteria‐derived antigens can rapidly and efficiently activate T cell in mLNs, promoting the generation of peripheral Tregs.^[^ ^15^ ^]^ Based on this, we hypothesized that *A. muciniphila* and its derived protein Amuc_1409 promote Tregs differentiation in mLNs and increase the proportion of peripheral Tregs. To test this hypothesis, we measured the percentage of CD4^+^CD25^+^Foxp3^+^‐Tregs in mLNs and found that treatment with *A. muciniphila*, Amuc_1409 or Amuc_1100 increased the proportion of Tregs in mLNs. Notably, *A. muciniphila* and Amuc_1409 induced a higher proportion of Tregs compared to Amuc_1100 (Figure 4H; Figure S6J, Supporting Information). To assess Treg infiltration in pancreatic tissue, we performed indirect immunofluorescence staining to analyze CD3 and Foxp3 expression in PBS‐treated and Amuc_1409‐treated SAP mice. Quantitative analysis revealed a marked increase in CD3⁺Foxp3⁺ double‐positive cells and distinct localization of Amuc_1409 in Amuc_1409‐treated mice compared to SAP controls (Figure S7A,B, Supporting Information), demonstrating that Amuc_1409 significantly promotes Treg recruitment to pancreatic tissue.

Then, we examined the effects of Amuc_1409 or Amuc_1100 on intestinal barrier and found that the gene expression of markers for tight junction, Muc2, Ocln, and Zo‐1, was significantly upregulated in colon of Amuc_1409 treated SAP mice (Figure S7C, Supporting Information). Consistent with the gene expression changes, the protein expression of Ocln and Muc2 was also increased in colon (Figure 4I—K; Figure S7D, Supporting Information) and duodenum (Figure S7E, Supporting Information) of Amuc_1409 treated SAP mice. Further, His‐tagged proteins were observably expressed in colon and duodenum of Amuc_1100 or Amuc_1409 treated mice. Previous studies have demonstrated that the Th17/Treg imbalance in SAP correlates with disease severity and poor prognosis,^[^ ^16^ ^]^ and Th17/Treg ratio is important for the intestinal barrier. To investigate this relationship, we isolated colon tissues from SAP model mice and analyzed single‐cell suspensions for Th17 and Treg proportions. Results demonstrated that compared to NC mice, SAP mice exhibited a significant reduction in colonic Treg populations and elevated Th17 cells populations. Amuc_1409 treatment normalized this imbalance, increasing Treg proportions, and reducing Th17 cells populations. The Th17/Treg ratio in Amuc_1409‐treated mice approached that of NC, contrasting sharply with untreated SAP mice (Figure S7F,G, Supporting Information)). These results suggest that Amuc_1409 maybe ameliorate intestinal barrier dysfunction by resolving Th17/Treg imbalance.

Collectively, these results suggest that Amuc_1409 protects the host from SAP by dampening pancreatic and systemic inflammation and promoting intestinal barrier integrity.

### 2.5 *Akkermansia Muciniphila* and Amuc_1409 Protect the Host from SAP in a Treg‐and IL‐10‐Dependent Manner

**Figure 5:**
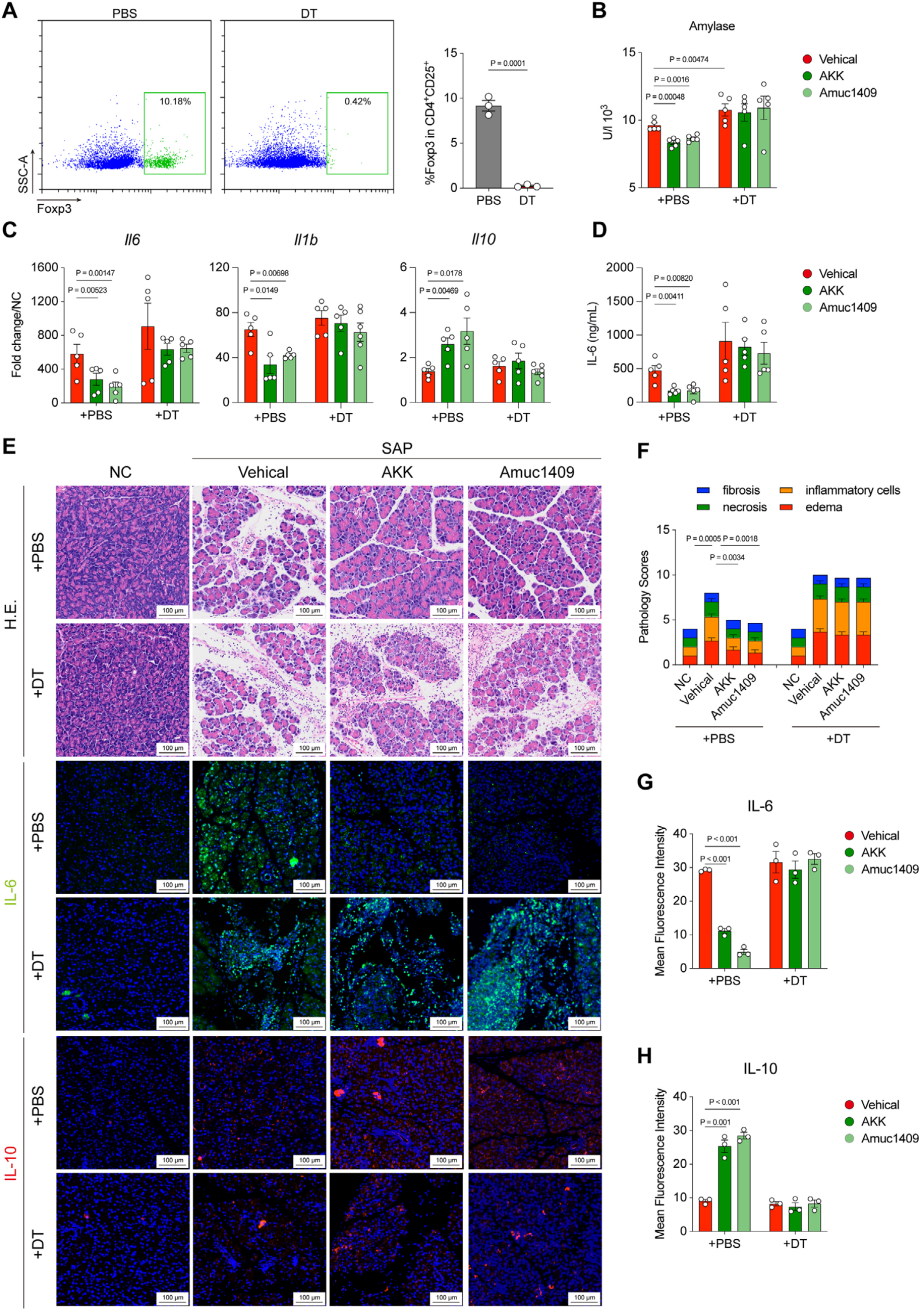
*A. muciniphila* and Amuc_1409 protect the host from SAP in a Treg‐dependent manner. Foxp3‐DTR mice were intraperitoneally injected with DT daily for 5 days, and splenic CD4^+^/CD25^+^/Foxp3^+^‐Tregs were detected by flow cytometry. A) Representative flow cytometry plots (left), and the percentage of CD4^+^/CD25^+^/Foxp3^+^‐Tregs (right) (*n* = 3). AKK or Amuc_1409 were gavaged to Tregs‐depletion mice and induced SAP using caerulein. B) Serum amylase level (*n* = 5), C) pancreatic *Il6, Il1b, Il10* mRNA level (*n* = 5), and D) serum IL‐6 level (*n* = 5), E) representative pancreatic histopathology (upper), IFA images of pancreatic IL‐6 (middle) and IL‐10 (down), F) pathology scores (*n* = 3), G,H) mean fluorescence intensity of (G) IL‐6 and (H) IL‐10. The two‐sided *p‐*values were examined by one‐way ANOVA with Dunnett's multiple comparisons test and data were presented as mean ± sem (A–D, F–H).

To verify whether *A. muciniphila* and Amuc_1409 protect the host from SAP through Tregs, we depleted Tregs in Foxp3‐DTR mice by intraperitoneal injection of diphtheria toxin (DT), and confirmed a significant reduction of CD4^+^CD25^+^Foxp3^+^‐Tregs in DT‐treated mice (**Figure** **5**A). Then, we gavaged *A. muciniphila* or Amuc_1409 to Tregs‐depletion mice and induced SAP using caerulein, and found that *A. muciniphila* or Amuc_1409 significantly reduced serum amylase content, pancreatic inflammatory cytokine *Il6*, *Il1b* mRNA level and serum IL‐6 expression, and upregulated pancreatic anti‐inflammatory cytokine *Il10* mRNA level in PBS‐treated Foxp3‐DTR mice, whereas *A. muciniphila* or Amuc_1409 did not alleviate serum amylase and pancreatic and systemic inflammatory responses in DT‐treated Foxp3‐DTR mice (Figure 5B–D). Moreover, *A. muciniphila* or Amuc_1409 treatment remarkedly alleviated pancreatic edema, necrosis, and inflammatory cells infiltration in PBS‐treated Foxp3‐DTR mice, but *A. muciniphila* or Amuc_1409 did not alleviate SAP induced pancreatic pathological lesion in DT‐treated Foxp3‐DTR mice (Figure 5E,F). Notably, Tregs depletion significantly aggravated pancreatic necrosis and inflammatory cells infiltration (Figure 5E,F). We further analyzed the pancreatic IL‐6 and IL‐10 expression through IFA assay, and found that *A. muciniphila* or Amuc_1409 significantly downregulated pancreatic IL‐6 expression and upregulated IL‐10 expression in PBS‐treated Foxp3‐DTR mice, whereas *A. muciniphila* or Amuc_1409 did not regulate the expression of IL‐6 and IL‐10 in DT‐treated Foxp3‐DTR mice (Figure 5E,G,H). Furthermore, we found that in PBS‐treated Foxp3‐DTR mice, *A. muciniphila* or Amuc_1409 administration significantly ameliorated colonic injury, with preserved epithelial integrity. However, in DT‐treated Foxp3‐DTR mice, the therapeutic effects of *A. muciniphila* or Amuc1409 were abolished (Figure S8A, Supporting Information), indicating a Treg‐dependent mechanism underlying their protective action on intestinal repair.

As a pivotal yet complex anti‐inflammatory cytokine, IL‐10 exhibits paradoxical effects in SAP patients–while some studies report significant elevation of IL‐10 levels,^[^ ^17^ ^]^ others demonstrate marked reduction.^[^ ^18^ ^]^ We hypothesize that this phenomenon may reflect dynamic equilibrium between pro‐inflammatory and anti‐inflammatory mediators during disease progression. To further elucidate IL‐10′s role in pancreatitis pathogenesis, we established SAP models using IL‐10 knockout (KO) mice. The IL‐10‐KO mice manifested exacerbated inflammatory responses and heightened pancreatic tissue damage compared to wild‐type controls (Figure S8B—E, Supporting Information), suggesting that IL‐10 deficiency may serve as a critical factor contributing to aggravated SAP pathology. To verify whether Amuc_1409 protect the host from SAP through IL‐10, we gavaged Amuc_1409 to IL‐10‐KO mice and induced SAP using caerulein, and found that Amuc_1409 significantly reduced serum amylase content, pancreatic inflammatory cytokine *Il6, Il1b* mRNA level and serum IL‐6 expression, and alleviated pancreatic edema, necrosis and inflammatory cells infiltration in WT mice, whereas Amuc_1409 did not alleviate serum amylase, pancreatic and systemic inflammatory responses and pancreatic damage in IL‐10‐KO mice (Figure S8B–E, Supporting Information).

Overall, these results suggest that the ameliorating effect of *A. muciniphila* and Amuc_1409 on SAP severity is mainly due to the increase of peripheral Tregs and IL‐10. Depletion of Tregs and IL‐10 discourage the protection of *A. muciniphila* or Amuc_1409 on SAP.

### 2.6 Amuc_1409 Promoted Tregs Differentiation and IL‐10 Production through Interaction with Ube2k

**Figure 6:**
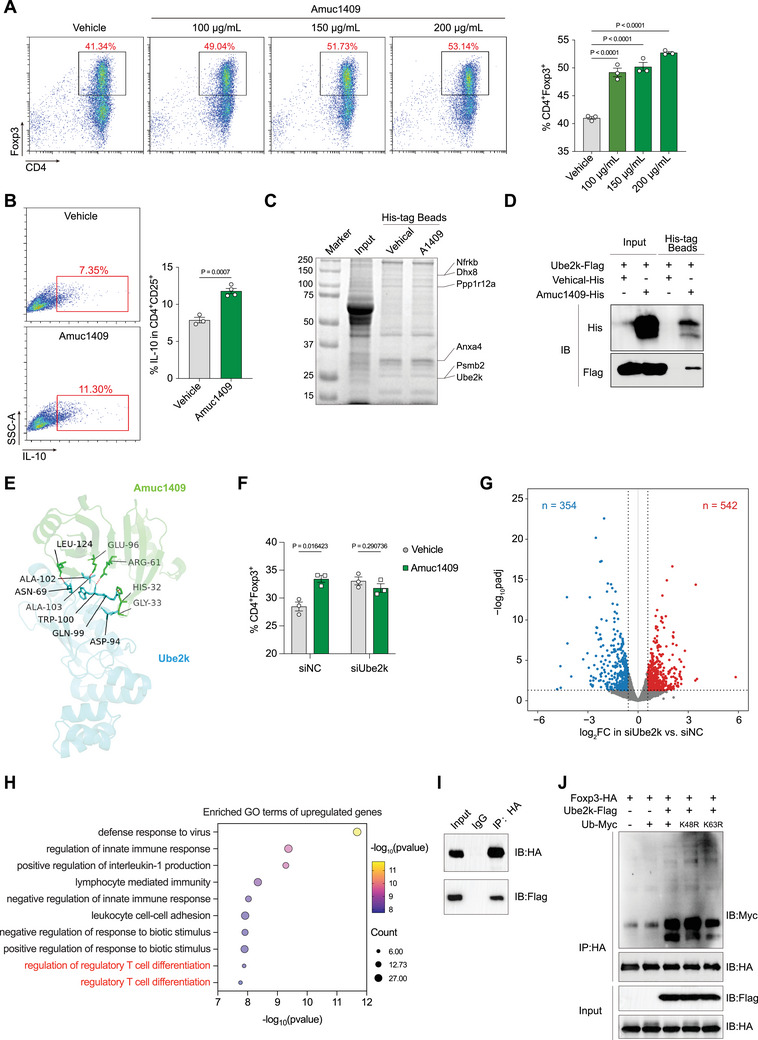
Amuc_1409 promoted Tregs differentiation and IL‐10 production through interaction with Ube2k. A) Mice naïve CD4^+^ T cells were isolated from spleen, and differentiated to Tregs via treating with or without different does of Amuc_1409 under IL‐2 and TGF‐β condition. Representative flow cytometry plots (left), and the percentage of CD4^+^/Foxp3^+^‐Tregs (right) (*n* = 3). B) Differentiated Tregs were treated with or without different does of Amuc_1409 under IL‐2 and TGF‐β condition. Representative flow cytometry plots (left), and the percentage of CD4^+^/CD25^+^/IL‐10^+^‐cells (right) (*n* = 3). C) Representative SDS‐PAGE analysis of binding complexes in Tregs extracts, incubated with or without His‐tagged Amuc_1409. D) HEK293T cells were co‐transfected with pcDNA3.1‐Amuc_1409‐His and pcDNA3.1‐Ube2k‐Flag. Co‐Immunoprecipitation assay was performed at 48 ho post transfections. Representative Western blot. E) Stimulation of the Amuc_1409 (Green)‐Ube2k (Blue) interaction. F) Differentiated Tregs were transfected with siNC or siUbe2k, and treated with or without 200 µg/mL of Amuc_1409 under IL‐2 and TGF‐β condition, the percentage of CD4^+^/Foxp3^+^‐Tregs (*n* = 3). G,H) Transcriptome analysis of differentiated Tregs transfected with siNC or siUbe2k. G) Volcano plot, H) Bubble plots of GO enrichment in upregulated genes in siUbe2k vs siNC transfected Tregs. I) HEK293T cells were co‐transfected with pcDNA3.1‐Foxp3‐HA and pcDNA3.1‐Ube2k‐Flag. Co‐Immunoprecipitation assay was performed at 72 h post transfections. Representative Western blot. J) HEK293T cells were co‐transfected with the indicated combinations of expression constructs encoding Foxp3‐HA, Ube2k‐Flag, and Myc‐tagged ubiquitin molecules—either wild‐type (Ub‐Myc) or a variant with a K‐to‐R mutation at either lysine residue 48 (K48R) or 63 (K63R) responsible for preventing K48‐ and K63‐type polyubiquitination, respectively. Co‐Immunoprecipitation assay was performed at 72 h post transfections. Representative Western blot. The two‐sided *p‐*values were examined by Student's *t*‐test (B) or one‐way ANOVA with Dunnett's multiple comparisons test (A, F) and data were presented as mean ± sem.

To investigate whether Amuc_1409 modulates macrophage‐mediated inflammatory responses, we conducted in vitro experiments using bone marrow‐derived macrophages (BMDMs) from C57BL/6 mice. Notably, Amuc_1409 treatment failed to attenuate LPS‐induced inflammatory activation in BMDMs within our experimental system (Figure S9A, Supporting Information). To examine the effect of Amuc_1409 on Tregs differentiation in vitro, we isolated mouse naïve CD4^+^ T cells, and differentiated them to Tregs via treating with or without Amuc_1409 under IL‐2 and TGF‐β condition. There was a significantly increase in the proportion of CD4^+^/Foxp3^+^‐Tregs under Amuc_1409 treatment, and in a dose‐dependent manner (**Figure** **6**A). We next assessed whether dendritic cells (DCs) were required for Amuc_1409‐mediated potentiation of Tregs differentiation. Murine bone marrow‐derived DCs (BMDCs) were induced by M‐CSF and IL‐4 in vitro, stimulated with Amuc_1409 or LPS (Positive control), and cocultured with Naïve CD4^+^ T cells to assess Tregs differentiation. Flow cytometry results revealed LPS, not Amuc_1409 stimulation significantly upregulated the proportion of CD11C^+^/F4/80^+^‐DCs (Figure S9B, Supporting Information), and Amuc_1409‐stimulated BMDC did not significantly enhanced Tregs differentiation, compared with vehicle‐treated BMDC (Figure S9C, Supporting Information), suggesting that Amuc_1409 promotes Tregs differentiation in a DCs‐independent manner.

To further test the role of Amuc_1409 on Tregs proliferation in vitro, we differentiated naïve CD4^+^ T cells to Tregs, and then treated with or without Amuc_1409. Flow cytometry results revealed Amuc_1409 remarkedly upregulated the proportion of CD4^+^/Foxp3^+^‐Tregs in a dose‐dependent manner (Figure S9D, Supporting Information). It suggests that Amuc_1409 promotes Tregs proliferation in vitro. In addition, we analyzed anti‐inflammatory cytokine IL‐10 expression of Tregs using flow cytometry. Amuc_1409 treatment significantly increased the IL‐10 expression of Tregs (Figure 6B). We further determined mRNA levels of inflammatory cytokines and anti‐inflammatory cytokines in Tregs using qPCR. Treatment with Amuc_1409 observably decreased the mRNA levels of *Il1b, Il6*, whereas increased the expression of *Foxp3, Il10* (Figure S9E, Supporting Information), implying that Amuc_1409 enhances anti‐inflammatory properties of Tregs.

Then, we investigated the underlying mechanism by which Amuc_1409 promotes Tregs differentiation and proliferation to induce IL‐10 expression. We first observed the subcellular localization of Amuc_1409 in Tregs via IFA, and found that Amuc_1409 mainly localized in the cytoplasm (Figure S10A, Supporting Information), suggesting that Amuc_1409 may enter cells and promote Tregs differentiation by interacting with certain proteins. Subsequently, by performing an in vitro binding assay to identify the proteins interacted with Amuc_1409 in Tregs via label‐free quantitative proteomics, we found that Amuc_1409 interacted with a range of proteins, such as, proteasome subunit beta type‐2 (Psmb2), ubiquitin‐conjugating enzyme E2 K (Ube2k), ATP‐dependent RNA helicase DHX8 (Dhx8), nuclear factor related to kappa‐B‐binding protein (Nfrkb), annexin A4 (Anxa4), protein phosphatase 1 regulatory subunit 12A (Ppp1r12a), and so on (Figure 6C). Among them, Psmb2 and Ube2k exhibited the highest protein abundance. Hence, we examined the interaction of these two proteins to Amuc_1409 through Co‐immunoprecipitation (Co‐IP) assay, and found that Amuc_1409 interacted with Ube2k but not Psmb2 (Figure 6D; Figure S10B, Supporting Information). Furthermore, we stimulated the Amuc_1409‐Ube2k interaction at the molecular level using AlphFold3, and found that Amuc_1409 interacted with six major amino acids (Asn^69^, Asp^94^, Gln^99^, Trp^100^, Ala^102^, and Ala^103^) from UBC core domain of Ube2k (Figure 6E). To further investigate whether Amuc_1409 promotes Tregs differentiation through interacting with Ube2k, we knocked down Ube2k expression of Tregs transfecting small interfering RNA (siRNA) (Figure S10C, Supporting Information), and then treated with or without Amuc_1409 to determine the differentiation of Tregs using Flow cytometry. We found that Amuc_1409 enhanced the differentiation of Tregs transfecting with siNC, whereas Amuc_1409 lose the ability to promote Tregs differentiation when transfected with siUbe2k. In addition, knocking down Ube2k expression promoted differentiation of Tregs with or without Amuc_1409 treatment (Figure 6F; Figure S10D, Supporting Information). Taken together, these results suggests that Amuc_1409 enhances Tregs differentiation and IL‐10 production through interaction with Ube2k.

To further investigate the contribution of Ube2k on regulation of Tregs differentiation, we performed RNA‐seq transcriptome profiling for Tregs transfected with siNC or siUbe2k. Differential expression analysis between siUbe2k and siNC‐transfected Tregs revealed 542 upregulated genes (log_2_ fold change > 0.585, adjusted *p*‐value < 0.05) and 354 downregulated genes (log_2_ fold change < −0.585, adjusted *p*‐value < 0.05) (Figure 6G). Gene Ontology (GO) enrichment analysis indicated that upregulated differentially expressed genes were mainly involved in terms such as regulation of regulatory T cell differentiation and regulation T cell differentiation (Figure 6H), and downregulated genes were involved in negative regulation of growth (Figure S10E, Supporting Information). Kyoto Encyclopedia of Genes and Genomes (KEGG) pathway enrichment analysis revealed upregulated genes in Tregs‐related pathways like Th17 cell differentiation (Figure S10F, Supporting Information), and downregulated genes were mainly enriched in JAK‐STAT signaling pathway (Figure S10G, Supporting Information). Taken together, knockdown Ube2k of Tregs exhibited a higher enrichment of Tregs and T cells differentiation and cells growth related signaling.

As a ubiquitin‐conjugating enzyme, Ube2k is capable of catalyzing the formation of ubiquitin chains with diverse linkage types, including K48‐linked and K63‐linked.^[^ ^19^ ^]^ Stable expression of Foxp3 is essential for both the generation and suppressive function of Tregs.^[^ ^20^ ^]^ However, Foxp3 is subjected to K48‐linked polyubiquitination, which mediates its degradation via the proteasome.^[^ ^21^ ^]^ Therefore, we hypothesized that Ube2k may mediate Foxp3 degradation via ubiquitination modification. To test this, we performed Co‐IP assays with the lysates of HEK293T cells transfected with expression vectors encoding HA‐tagged Foxp3 and Flag‐tagged Ube2k. Upon immunoprecipitation of Foxp3, we detected Ube2k protein in the material recovered from lysate (Figure 6I), indicating that Ube2k interacts with the Treg transcription factor Foxp3. Next, Co‐expression of Foxp3‐HA and Ube2k‐Flag constructs, along with distinct Myc‐tagged ubiquitin variants, allowed us to confirm the nature of the Foxp3 modification arising from this interaction. Pull‐down of Foxp3‐HA protein from cell lysates by anti‐HA antibody enabled us to examine the various ubiquitinated forms of Foxp3 by immunoblot analysis. We observed substantial ubiquitination of Foxp3 in cells triple‐transfected with expression constructs encoding Foxp3, Ube2k, and wild‐type ubiquitin (Figure 6J). Interestingly, the expression of ubiquitin molecules with a mutated lysine residue at K48 (K48R), which cannot support K48 polyubiquitination, led to a robust Foxp3 ubiquitination in this system. In contrast, expression of a K63R ubiquitin mutation resulted in a significant reduction of Foxp3 ubiquitination (Figure 6J). These results suggest that Ube2k facilitates a K63‐type ubiquitination of Foxp3.

### 2.7 Patients with SAP Exhibit a Decreased Percentage of Tregs that was Inversely Correlated with Fecal Genomic Copies of *A. Muciniphila*

**Figure 7:**
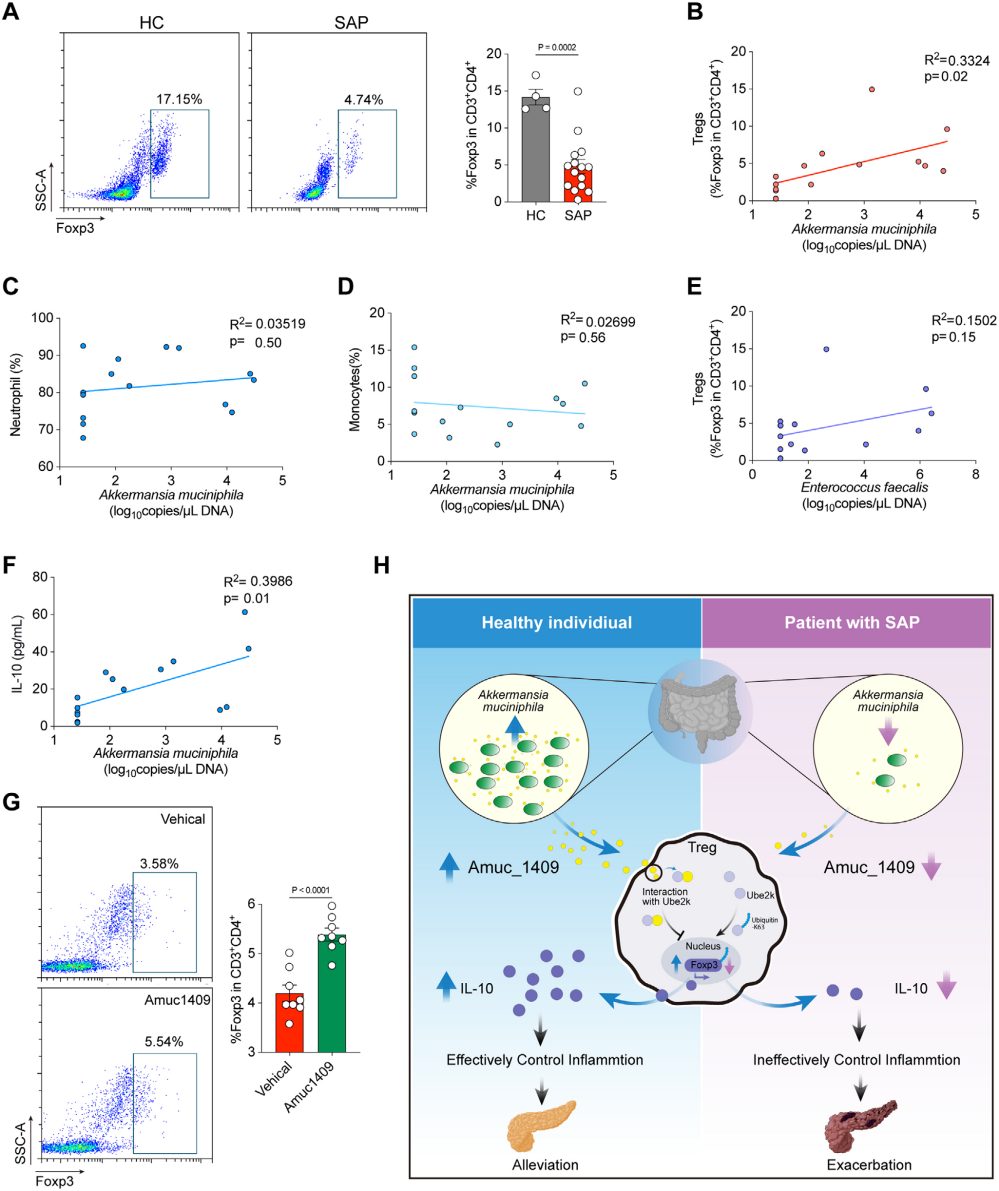
Amuc_1409 in regulating the population of Tregs in PBMCs from patients with SAP. A) The PBMCs of healthy individuals and patients with SAP were isolated, and the population of peripheral CD3^+^/CD4^+^/Foxp3^+^‐Tregs analyzed by flow cytometry. Representative flow cytometry plots (left), and the percentage of CD3^+^/CD4^+^/Foxp3^+^‐Tregs (right) (*n* = 4–15). B–F) The percentages of Tregs, Neutrophils, and Monocytes were determined in PBMCs of patients with SAP, and the genomic copies of *A. muciniphila* and *Enterococcus fecalis* were determined in feces of patients with SAP. B–D) Dot plot showing the correlation of genomic copies of *A. muciniphila* and percentages of Tregs (B), Neutrophils (C), or Monocytes (D). E) Dot plot showing the correlation of percentages of Tregs and genomic copies of *E. fecalis*, and F) the correlation of concentration of IL‐10 and genomic copies of *A. muciniphila*. G) The PBMCs of healthy individuals and patients with SAP were isolated, then treated with or without 200 µg mL^−1^ Amuc_1409 and analyzed the population of peripheral CD3^+^/CD4^+^/Foxp3^+^‐Tregs by flow cytometry. Representative flow cytometry plots (left), and the percentage of CD3^+^/CD4^+^/Foxp3^+^‐Tregs (right) (*n* = 8). H) Schematic illustration of *A. muciniphila* alleviates severe acute pancreatitis via Amuc1409‐ube2k‐foxp3 axis in Tregs. The two‐sided *p‐*values were examined by Student's *t*‐test and data were presented as mean ± sem (A,G). *R^2^* and exact two‐sided *p‐*values calculated by Pearson's test are shown (B–F).

Finally, we isolated the PBMCs of healthy individuals and patients with SAP, and analyzed the population of peripheral Tregs by flow cytometry. The population of peripheral Tregs were significantly reduced in PBMC of patients with SAP, compared with healthy control (**Figure** **7**A). Subsequently, we quantified the fecal genomic copies of *A. muciniphila* and *E. faecalis*, and analyzed their correlations with the populations of peripheral Tregs, neutrophils, and monocytes. Our results revealed a positive correlation between the fecal genomic copies of *A. muciniphila* and the population of peripheral Tregs (Figure 7B). However, no such correlation was observed with the populations of peripheral neutrophils (Figure 7C) or monocytes (Figure 7D). Additionally, the population of peripheral Tregs showed no correlation with the fecal genomic copies of *E. faecalis* (Figure 7E), but the serum concentrations of IL‐10 showed a positive correlation with the fecal genomic copies of *A. muciniphila* (Figure 7F). Collectively, these correlation analysis data imply that *A. muciniphila* might mitigate inflammatory responses in patients with SAP by augmenting the population of peripheral Tregs.

To explore the therapeutic efficacy of Amuc_1409 in patients with SAP, PBMCs were isolated from these patients and then treated either with or without Amuc_1409. Subsequently, the population of Tregs was analyzed via flow cytometry. Notably, treatment with Amuc_1409 led to a significant increase in the population of Tregs within the isolated PBMCs from SAP patients (Figure 7G). These findings validate the function of Amuc_1409 in modulating the Treg population in PBMCs obtained from SAP patients. Moreover, they suggest that in vivo administration of Amuc_1409 could potentially exert therapeutic benefits in patients with SAP by upregulating the Treg population, thereby suppressing inflammation.

## 3 Discussion

In this study, we elucidated an *A. muciniphila*–Amuc_1409–Ube2k axis that enhances the anti‐inflammatory responses mediated by peripheral Tregs, thereby mitigating host SIRS associated with SAP and potentially other systemic inflammatory diseases (Figure 7H). The patients with AP exhibited a significantly lower abundance of fecal *Akkermansia*, which was also reduced with the severity of AP, suggesting a potential role of *Akkermansia* as a microbial biomarker for outcome of AP. One limitation of this study was the inability to collect stool samples from patients with AP before they show clinical symptoms. Consequently, we cannot determine whether the decrease in fecal *Akkermansia* abundance was a result of AP itself, or if inherent differences in fecal *Akkermansia* levels among patients contributed to varied AP outcomes. This raises the question of whether patients with lower baseline fecal *Akkermansia* abundance might be more susceptible to severe conditions. However, in our previous mouse model, we observed a significant reduction in fecal *Akkermansia* abundance in MAP mice compared to PBS controls,^[^ ^9^ ^]^ suggesting that AP may indeed contribute to a decrease in *Akkermansia* abundance. In another disease involving severe fever with thrombocytopenia syndrome virus (SFTSV) infection‐induced systemic inflammatory response, we also observed a similar phenomenon, where changes in the abundance of *Akkermansia* caused by the infection affected the disease outcome.^[^ ^22^ ^]^

Recent studies have highlighted the role of *A. muciniphila* in reducing systemic inflammatory responses. Research shows that *A. muciniphila* mitigates systemic inflammation through its metabolite harmaline, which acts via the bile acid‐G‐protein‐coupled receptor‐5 (TGR5)‐NF‐kB signaling.^[^ ^22^ ^]^ Furthermore, *A. muciniphila* secreted threonyl‐tRNA synthetase (*Am* TARS) targets macrophages and activates the anti‐inflammatory TLR‐CREB axis, leading to increase IL‐10 production and attenuates colitis in mice.^[^ ^23^ ^]^ In this study, we observed that the protein components of *A. muciniphila* demonstrate superior efficacy compared to its metabolites in attenuating both pancreatic and systemic inflammatory responses. Therefore, our study primarily focuses on the regulatory effects of *A. muciniphila*‐derived proteins, rather than metabolites, on AP. However, this focus does not preclude the potential regulatory role of *A. muciniphila*‐derived metabolites such as *Am* TARS in AP pathogenesis. Notably, Amuc_1100, an outer membrane protein of *A. muciniphila*, plays a crucial role in immune regulation and intestinal barrier function by activating TLR2 and TLR4, subsequently increasing IL‐10 production.^[^ ^13^, ^24^ ^]^ Recent literature also highlights the role of Amuc_1100 in acute pancreatitis, with findings indicating that Amuc_1100 pretreatment alleviates AP by reducing macrophage and neutrophil infiltration and modulating gut microbiota composition and tryptophan metabolism.^[^ ^10^ ^]^ Consistent with these findings, our study demonstrates that Amuc_1100 effectively mitigates SAP‐induced inflammatory responses and pancreatic immunopathological injury. This study revealed that another key protein, Amuc_1409, significantly reduced pancreatic and systemic inflammatory responses, thereby alleviated pancreatic immunopathological injury. Amuc_1409 is recognized as one of the most abundant secreted proteins, capable of localizing within the periplasmic space and outer membrane before export to the extracellular space.^[^ ^25^ ^]^ Additionally, Amuc_1409 exhibits high thermal stability, similar to that of Amuc_1100.^[^ ^12^, ^13^ ^]^ Future studies are required to develop a mutant *A. muciniphila* strain with silenced Amuc_1100 and/or Amuc_1409 genes to determine whether *A. muciniphila's* protective effects against SAP are mediated through Amuc_1100 and/or Amuc_1409. Currently, there have been no reported side effects associated with Amuc proteins. However, it is conceivable that prolonged or high‐dose administration of proteins such as Amuc_1100 may excessively activate Toll‐like receptors (TLRs) in vivo, leading to systemic immune overactivation, cytokine storms, or immune dysregulation. Consequently, further investigations are required to clarify the appropriate dosage, frequency, and potential adverse effects of these immunomodulatory proteins.

Using Foxp3‐DTR mice, we verified that the protective effects of *A. muciniphila* and its secreted protein Amuc_1409 against SAP in mice depend on the presence of Tregs, underscoring the critical role of Tregs in SAP. Recent studies have shown that while preventive depletion of Tregs enhances inflammatory responses, it concurrently stabilizes the immune barrier functions of Th17 cells and CD8^+^γδTCR^+^ intestinal epithelial cells, significantly reducing the translocation of intestinal bacteria to the pancreas.^[^ ^26^ ^]^ However, our results indicate that the increase in Tregs in PBMCs helps alleviate inflammation induced by SAP, seemingly presenting contradictory conclusions. This discrepancy arises from the distinct roles Tregs play in different organs during AP. In the intestine, Tregs disrupt the immune barrier functions of Th17 cells and CD8^+^γδTCR^+^ intestinal epithelial cells, increasing the likelihood of bacterial translocation from the gut to the pancreas. The referenced study used a bile duct ligation‐induced acute pancreatitis model, which elicited a relatively mild inflammatory response in mice. Thus, Tregs with immunosuppressive functions did not inhibit the body's inflammatory response but rather weakened the intestinal immune barrier, exacerbating bacterial translocation. In contrast, our SAP model, induced by cerulein combined with LPS or arginine combined with LPS, generated a robust initial inflammatory response. As a result, the Tregs induced by *A. muciniphila* and Amuc_1409 could rapidly suppress excessive inflammation, thereby alleviating SAP in mice. This dual role of Treg function presents a critical therapeutic challenge: the precise timing and dosage of *A. muciniphila* or its driven protein Amuc_1409 administration must be carefully optimized to balance their beneficial anti‐inflammatory effects in early AP against potential risks of immunosuppression in later stages. Addressing this challenge will be a major focus of our future research.

During the initial phase of SAP or sepsis, a massive release of pro‐inflammatory cytokines (e.g., IL‐6, TNF‐α) establishes a critical immunoregulatory imbalance. Elevated IL‐6 levels suppress Treg differentiation and survival through STAT3 signaling pathway activation, while concurrently driving Th17 cell expansion.^[^ ^27^ ^]^ This imbalance leads to a transient depletion of Tregs as the immune system prioritizes pro‐inflammatory responses, potentially through Treg functional inhibition or apoptosis. Our experimental design specifically targets this early inflammatory window, with sample collection timed to capture the cytokine storm phase, aiming to develop strategies that enhance Treg‐mediated immunosuppression to rapidly terminate pathological hyperinflammation. Notably, excessive Treg activation may paradoxically induce the CARS, predisposing patients to infectious pancreatic necrosis during later disease stages. This biphasic immunopathology underscores the therapeutic challenge of precisely regulating Treg induction timing and activation intensity. Optimal intervention requires: stage‐specific modulation of Treg plasticity, threshold‐controlled immunomodulatory signals, and microenvironment‐adapted functional programming. These parameters constitute critical foci for our ongoing investigations into SAP pathophysiology and targeted immunotherapy development.

Through in vitro pull‐down and Co‐IP experiments, we confirmed Amuc_1409 interacts with the ubiquitin‐conjugating enzyme Ube2k, promoting Treg differentiation, proliferation, and anti‐inflammatory IL‐10 expression in a Ube2k‐dependent manner. Ube2k, an E2 ubiquitin‐conjugating enzyme, plays an important role in gene regulation and cellular function. Recent studies have shown that Ube2k binds to histone H3, induces its ubiquitination, and regulates neural stem cell differentiation and proliferation through proteasome‐mediated degradation of histone H3.^[^ ^28^ ^]^ Additionally, Ube2k promotes the proliferation and migration of hepatocellular carcinoma cells by upregulating c‐Myc, while knockdown of Ube2k significantly inhibits the malignancy of liver cancer.^[^ ^29^ ^]^ These findings highlight Ube2k's critical regulatory role in cell differentiation and proliferation. Our results demonstrate that Ube2k directly interacts with Foxp3 and promotes its K63‐linked polyubiquitination, which may lead to protein destabilization of this critical transcriptional regulator of Treg cell differentiation and IL‐10 production. Given Foxp3's pivotal role in maintaining Treg populations and anti‐inflammatory responses, Ube2k‐mediated ubiquitination could consequently reduce Treg numbers and diminish the transcription of key anti‐inflammatory factors. Importantly, we propose that Amuc_1409 may exert its protective effects by binding to Ube2k and competitively inhibiting Foxp3 ubiquitination, thereby preserving Foxp3 stability and promoting Treg differentiation along with IL‐10 expression. Future studies employing genetic manipulation approaches (e.g., CRISPR‐Cas9‐mediated knockout or overexpression of Ube2k in Tregs) would provide mechanistic validation of its role in Amuc_1409‐induced Treg cell differentiation.

In conclusion, this study demonstrates that Amuc_1409, derived from *A. muciniphila*, plays a crucial role in suppressing SAP‐induced inflammation through its direct interaction with the Ube2k protein in Tregs. This work advances the understanding of microbiota‐host interactions mediated by bioactive molecules. More importantly, it opens up potential avenues for developing a new probiotic product aimed at improving acute pancreatitis and other inflammatory disorders.

## 4 Experimental Section

### Ethics Statement

The experimental protocols were approved by the Institutional Animal Care and Use Committee of Sir Run Run Shaw Hospital (No. 202 402 078). All methods were carried out in accordance with relevant guidelines and regulations. This study was conducted as recommended by the ARRIVE guidelines.

### Patients and Sample Collection

A total of 86 hospitalized patients (38 with MAP, 16 with MSAP, and 32 with SAP) and 46 healthy control volunteers (HC), aged between 20 and 88 years old, were included in the study. These individuals, who exhibited symptoms within 7 days of onset, were recruited from the Intensive Care Unit at Sir Run Run Shaw Hospital, affiliated with Zhejiang University Medical College. All participants met the criteria for SAP as defined by the revised Atlanta classification. Faecal and serum specimens were collected from patients on admission. Specimens were stored in sterile EP tubes, and kept at −80 °C. Every participant provided a written informed consent, adhering to protocols approved by the ethical committees of Sir Run Run Shaw Hospital (No. 2025‐2053‐01).

### 16S rRNA Amplicon Sequencing and Data Analyses

≈200 mg of fecal samples were resuspended in Qiagen's ASL buffer and homogenized for 2 min. Total fecal DNA was extracted from the supernatant using the QIAamp DNA Stool Mini Kit (Qiagen). The concentration and purity of the extracted DNA were assessed using the Qubit fluorometer (Thermo Fisher Scientific).

For amplification, the stool DNA was subjected to PCR using the Phusion High‐Fidelity PCR Master Mix (New England Biolabs), targeting the V3–V4 variable regions of the 16S rRNA gene. The forward primer used was ACTCCTACGGGAGGCAGCA, and the reverse primer was GGACTACHVGGGTWTCTAAT. Amplicons, tagged with sample‐specific barcodes, were then sequenced using an Illumina NovaSeq platform.

Paired‐end reads from the sequencing were merged into longer sequences using FLASH v.1.2.7,^[^ ^30^ ^]^ a tool optimized for merging paired‐end reads with overlapping regions. The merged sequences were subsequently analyzed with the QIIME v.1.9.1 software package.^[^ ^31^ ^]^

### Animals, Bacteria, and Cell Culture

C57BL/6J mice were obtained from the Model Animal Research Center of Nanjing University (Nanjing, China). B6‐Il10‐KO mice (Strain NO. T005959) were purchased from GemPharmatech (Nanjing, China). All experiments were conducted using 6‐week‐old mice with an average weight of 20 ± 5 g. The mice were housed in a sterile environment under controlled conditions, with an average temperature of 22 °C and a standard 12 h light/dark cycle (7:00 am to 7:00 pm) at the animal experimentation center of Zhejiang University.

*Akkermansia muciniphila* (catalog no. BAA835) was obtained from the American Type Culture Collection (ATCC) and cultured in Brain Heart Infusion (BHI) medium (Oxoid) at 37 °C under anaerobic conditions. *Enterococcus faecalis* (catalog no. AB 2 018 154) was purchased from the CCTCC and cultured in lysogeny broth (LB) medium (Oxoid) at 37 °C. The concentration of each bacterial species was quantified by measuring the optical density at 600 nm (OD600).

HEK293T cells were purchased from ATCC and cultured in DMEM with 10% fetal bovine serum (FBS, Gibco). Human PBMCs were extracted by density‐gradient centrifugation using human PBMC isolation kits (TBD) following the manufacturer's protocol. After isolation, PBMCs were cultured in RPMI‐1640 medium supplemented with 10% FBS at 37 °C.

Naïve CD4^+^ T cells were isolated using EasySep Mouse Naïve CD4^+^ T Cell Isolation Kit (STEMCELL) following the manufacturer's protocol. After isolation, Naïve CD4^+^ T cells were plated to 1 µg mL^−1^ CD3/CD28 pretreated cell plates and cultured in RPMI‐1640 medium supplemented with 10% FBS, 1 ng mL^−1^ TGF‐β, 5 ng mL^−1^ IL‐2 at 37 °C. Tregs were harvested at 3 days post‐differentiation.

Isolation and in vitro induction of murine bone marrow‐derived dendritic cells (BMDCs) and murine bone marrow‐derived macrophages (BMDMs) were carried out as previously described.^[^ ^32^ ^]^ Briefly, the femurs and tibias were surgically excised and thoroughly washed with sterile phosphate‐buffered saline (PBS) to procure a cell suspension. Subsequently, red blood cells were lysed, and bone marrow cells (BM cells) were harvested by centrifugation. For induction of BMDCs, the BM cells were then plated at a density of 1 × 10^6^ cells mL^−1^ in 2 mL of cell culture medium supplemented with 20 ng mL^−1^ granulocyte‐macrophage colony‐stimulating factor (GM‐CSF) and 20 ng mL^−1^ interleukin‐4 (IL‐4) in the central area of 6‐well plates. For induction of BMDMs, the BM cells were then plated at a density of 1 × 10^6^ cells mL^−1^ in 2 mL of cell culture medium supplemented with 20 ng mL^−1^ GM‐CSF in the central area of 6‐well plates. On the third and fifth days of culture, half of the supernatant was carefully aspirated, and fresh cytokines were added at an equal volume. On Day 5, Amuc_1409 (at a concentration of 100 µg mL^−1^) or lipopolysaccharide (LPS, at a concentration of 1 µg mL^−1^) was incorporated into the culture media.

Foxp3‐DTR mice received intraperitoneal (i.p.) injections of 1 µg DT for 5 days to deplete Tregs. The efficiency of depletion was determined by Flow cytometry.

### Antibiotics (Abx) Treatment, Faecal Microbial Transfer, and Bacterial Colonization

Mice were administered an antibiotic cocktail consisting of ampicillin, neomycin, metronidazole, and vancomycin via oral gavage for 5 days, following previously described protocols.^[^ ^33^ ^]^ Antibiotics were also added to their drinking water, and the animals were maintained on either Abx‐containing or PBS‐containing water for the duration of the experiments.

For FMT experiments, fecal sample were collected from none‐treated mice, HC volunteers or patients with SAP and processed as described previously.^[^ ^34^ ^]^ In brief, fecal samples were weighted and homogenized with 1 mL sterile PBS, filtered through a 100 µm strainer, and centrifuged at 6000 g for 15 min. The pellets were resuspended in PBS with 10% (v/v) glycerol and stored at −80°C. Fecal sample were centrifuged and resuspended in sterile PBS, and gavaged to Abx‐treated mice with 20 mg fecal in 200 µL PBS at 6 d after Abx treatment.

For the bacterial colonization and FMT experiments, Abx mice were gavaged with 10^10^ CFU of *A. muciniphila*, *E. faecalis* or 20 mg FMT in 200 µL of PBS at 6 d after Abx treatment. After 48 h of colonization, SAP models were constructed.

### Bacterial Supernatant and Protein Components Preparation

*A. muciniphila* cell were centrifuged at 6000 g for 15 min, and the collected supernatant was centrifuged at 12 000 g for 15 min at 4 °C. Subsequently, the supernatant was passed through polyether‐sulfone filters (0.22 µm, Merck Millipore) to remove the residual bacterial cells. The supernatants were passed through 100, 50 and 10 kDa filters (Merck Millipore) at 3200 g for 10 min. Each intercepting fluid (including secretory proteins) was mixed with *A. muciniphila* cell, and then the mixture was inactivated by pasteurization for 30 min at 70 °C (Named p‐AKK). The filtrate passed 10 kDa filter were collected and named s‐AKK.

The mice gavaged with 200 µL s‐AKK or 10^9^ CFU p‐AKK were initiated in parallel with Abx treatment until the end of experiments.

### Expression and Purification of His‐Tagged Amuc_1409 and His‐Tagged Amuc_1100 Protein

The Amuc_1409 and Amuc_1100 genes were synthesized and subcloned into pET30a vectors using *E. coli* DH5α. The resulting plasmids, pET30a‐1409 and pET30a‐1100, were verified by DNA sequence analysis. The purified plasmids containing the recombinant Amuc_1409 and Amuc_1100 genes were transformed into *E. coli* BL21 (DE3). The transformed *E. coli* strains were grown in LB broth supplemented with kanamycin (50 µg mL^−1^) at 30 °C while shaking at 120 rpm. In the mid‐exponential phase, 0.5 mm IPTG was added to induce protein expression. After centrifugation at 6000 g for 20 min at 4 °C, the cells were harvested. Next, the cell pellets were resuspended in an ice‐cold buffer consisting of 300 mm NaCl, 50 mm Tris‐HCl (pH 8.0), and 10 mm imidazole. The cell suspensions were sonicated to obtain crude cell extracts. The crude cell extracts were further processed by centrifugation at 12 000 g for 30 min at 4 °C. The resulting cell lysates containing the 6xHis‐Amuc1409 and 6xHis‐Amuc1100 fused proteins were applied to Ni Sepharose 6 Fast Flow columns. Endotoxin was removed from the recombinant proteins using high‐capacity endotoxin removal spin columns according to the manufacturer's instructions. After buffer exchange using 7 MWCO zeba spin desalting columns (Thermo Fisher Scientific), the protein contents were determined using the BCA assay (Thermo Fisher Scientific). The purified Amuc_1409 and Amuc_1100 proteins were then stored at −80 °C.

The mice gavaged with Amuc_1409 or Amuc_1100 proteins (5 µg per mouse) were initiated in parallel with Abx treatment until the end of experiments.

### Induction of Severe Acute Pancreatitis Mouse Models

For the cerulein‐induced SAP model, a group of mice received seven intraperitoneal (i.p.) injections of cerulein (50 µg kg^−1^, MCE) at 1 h intervals, followed by a single i.p. injection of LPS (10 mg kg^−1^, Sigma) after the final cerulein injection. Samples were collected 12 h after the first cerulein injection.

For the cerulein‐induced MAP model, a group of mice received seven intraperitoneal (i.p.) injections of cerulein (50 µg kg^−1^, MCE) at 1 h intervals, after the final cerulein injection. Samples were collected 12 h after the first cerulein injection.

For the L‐arginine‐induced SAP model, mice were given two hourly i.p. injections of 10% L‐arginine (4 g kg^−1^, pH 7). Three days later, the mice received an i.p. injection of LPS (10 mg kg^−1^), and samples were collected 4 h after the initial LPS injection.

### Serum Amylase and Cytokine Expression Analysis

Serum amylase concentration was determined by the Alpha‐amylase Determination Kit (BIOSINO, Beijing, China) following the manufacturer's protocol.

Total RNA from bead‐homogenized tissue samples or cell culture was extracted using TRIzol reagent (Invitrogen) following the manufacturer's protocol. The cytokine level was determined by quantitative PCR with reverse transcription using the HiScript II One Step qRT–PCR SYBR Green Kit (Vazyme).

The following primers were used, GAPDH, forward, 5′‐GCCTTCCGTGTTCCTACCC‐3′; reverse, 5′‐CCCTCAGATGCCTGCTTCAC‐3′. IL‐6, forward, 5′‐AGGATACCACTCCCAACAGA‐3′; reverse, 5′‐ACTCCAGGTAGCTATGGTACTC‐3′. IL‐1β, forward, 5′‐ AAGCCTCGTGCTGTCGGACC‐3′; reverse, 5′‐ TGAGGCCCAAGGCCACAGGT‐3′. TNF‐α, forward, 5′‐CATCTTCTCAAAATTCGAGTGACAA‐3′; reverse, 5′‐ CCAGCTGCTCCTCCACTTG‐3′. UBE2K, forward, 5′‐CAGCGAATCAAGCGGGAGTT‐3′; reverse, 5′‐AGGTCCTGCTATTTCTCCTCTT‐3′.

IL‐6, TNF‐α, IL‐10 protein levels in serum samples were measured by the corresponding enzyme‐linked immunosorbent assay (ELISA) kits (MultiSciences) following the manufacturer's protocol.

### Fecal Bacteria Quantification

Fecal bacterial quantification was determined through qPCR. The fecal bacterial DNA was isolated with a TIANamp Stool DNA Kit (TIANGEN). SYBR Green Real‐time PCR Master Mix (TOYOBO) was used for qPCR.

The following primers were used, *Akkermansia muciniphila*, forward, 5′‐CAGCACGTGAAGGTGGGGAC‐3′; reverse, 5′‐CCTTGCGGTTGGCTTCAGAT‐3′. *Enterococcus fecalis*, forward, 5′‐CCGAGTGCTTGCACTCAATTGG‐3′; reverse, 5′‐CTCTTATGCCATGCGGCATAAAC‐3′.

### Tissue Histology and Staining

Pancreas, colon, and duodenum tissues were fixed in 4% paraformaldehyde, dehydrated, embedded in paraffin, and sectioned into 4 µm thick slices. These sections were then stained with hematoxylin and eosin (H&E) following standard protocols.

For immunohistochemistry, deparaffinized pancreas sections were blocked with 10% normal goat serum for 30 min and incubated overnight at 4 °C with an anti‐IL‐6 polyclonal antibody or an anti‐IL‐10 polyclonal antibody (Proteintech). The pancreas, spleen, colon, and duodenum were blocked with 10% normal goat serum for 30 min and incubated overnight at 4 °C with the anti‐ZO‐1 polyclonal antibody, anti‐OCLN polyclonal antibody, anti‐MUC‐2 polyclonal antibody, anti‐CD3 polyclonal antibody, anti‐Foxp3 polyclonal antibody, and anti‐His polyclonal antibody (Proteintech). The sections were subsequently incubated with a FITC/Cy3/Cy5‐conjugated anti‐rabbit secondary antibody for 55 min at room temperature. Finally, the sections were incubated with 4′,6‐diamidino‐2‐phenylindole (DAPI) solution for 10 min at room temperature. Fluorescence microscopy was performed, and images were captured accordingly.

### Isolation of Lymphocytes from Colon

Mice were euthanized by cervical dislocation, and the colon was aseptically excised. After removing Peyer's patches and adipose tissue, the intestine was longitudinally opened, washed with PBS, and cut into fragments. Tissue digestion was performed using a solution containing 1 mg mL^−1^ collagenase D and 1 mg mL^−1^ DNase I in RPMI‐1640 medium (37 °C, 40–60 min). The digested suspension was filtered through a 70 µm cell strainer, and single‐cell suspensions were enriched via Percoll gradient centrifugation (30 and 70% layers, 2000 rpm, 20 min). Cells at the 70–30% interface were collected, washed, and resuspended in staining buffer.

### Flow Cytometry Analysis

Flow cytometry analysis was performed on PBMCs, colonic lymphocytes, and splenocytes. The cells were separated through a 70 µm cell strainer. After washing and lysis of erythrocytes using red blood cell lysis buffer (solarbio), cells were preincubated with FcR blocking reagent (Biolegend) and stained for extracellular surface marker (CD4, CD25; CD11b, F4/80, Ly6G) at 4 °C for 20 min. For staining of Treg cells, the True‐Nuclear Transcription Factor Buffer Set (Biolegend) was used for fixation and permeabilization. Then, the cells were stained for transcription factors (Foxp3) at 4 °C for 60 min. For staining of Th17 cells, the Fixation Buffer and Intracellular Staining Perm Wash Buffer (Biolegend) were used for fixation and permeabilization. Then, the cells were stained for IL17A at 4 °C for 60 min. Data evaluation was performed by flow cytometry (CytoFLEX LX, BECKMAN).

### Single‐Cell Sequencing

scRNA‐seq was performed by Cosmos Wisdom Biotech Co., Ltd. In brief, the scRNA‐seq libraries were generated using the 10X Genomics Chromium Controller Instrument and Chromium Single Cell 3′ V3.1 Reagent Kits (10X Genomics, Pleasanton, CA). Briefly, cells were concentrated to 1000 cells uL^−1^ and ≈10 000 cells were loaded into each channel to generate single‐cell Gel Bead‐In‐EMulsions (GEMs), which resulted into expected mRNA barcoding of 5000 single‐cells for each sample. After the RT step, GEMs were broken and barcoded‐cDNA was purified and amplified. The amplified barcoded cDNA was fragmented, A‐tailed, ligated with adaptors and index PCR amplified. The final libraries were quantified using the Qubit High Sensitivity DNA assay (Thermo Fisher Scientific) and the size distribution of the libraries were determined using a High Sensitivity DNA chip on a Qsep100 (Bioptic). All libraries were sequenced by NovaSeq 6000 (Illumina, San Diego, CA) on a 150 bp paired‐end run.

### Single‐Cell RNA Statistical Analysis

Fastp^[^ ^35^ ^]^ was applied with default parameter filtering the adaptor sequence and removed the low quality reads to achieve the clean data. Then, the feature‐barcode matrices were obtained by aligning reads to the mouse genome using CellRanger v7.1.0 (10X Genomics). All scRNA‐seq reads were aligned to the mouse reference genome (mm10) and the cell‐by‐gene count matrices were produced by the CellRanger pipeline with default parameters. This process performed sequence alignment, filtering, label calculation, and unique molecular identifier (UMI) calculation. Finally, the gene file, tag file, and gene‐tag matrix file corresponding to each sample were generated.

The result files generated by CellRanger were processed using R package Seurat (v4.0.4) to individually integrate each sample and generate Seurat objects. The filtration criteria for low quality cells were as follows: 1) The abnormally total number of genes. 2) The abnormally total number of transcripts. 3) The high expression percentage of mitochondrial genes, ribosomal genes, heat‐shock genes, and dissociated genes in each cell. The quickPerCellQC function in the R package scater (v1.18.6) was used to filter low quality cells. The nmad parameter in the function quickPerCellQC represented the median absolute deviation (MAD, in this study nmad = 3). Cells that meet one of the above quality control criteria were considered to be of low quality and were filtered out.

After quality control of each sample, the Seurat objects of all samples were integrated, and the Seurat package was used to perform standard process on the merged objects for subsequent analysis. Specifically, variable genes were found using the FindVariableFeatures function with parameters: selection.method = “vst”, nfeatures = 500, and the ScaleData function was used to regress out variation due to differences in total UMIs per cell. Principal component analysis was used on the scaled expression profiles for the variable genes, and the top 20 principal components (PCs) were conducted for cell clustering. Cells were clustered in PCA space using Seurat's FindNeighbors on the top 30 PCs, followed by shared nearest neighbor modularity optimization‐based clustering algorithm. All cells were visualized by uniform manifold approximation and projection (UMAP). Cell subtypes were classified and annotated by examining differentially expressed genes in each cell subtype and observing the expression of specific marker genes of known cell types collected from publications. The FindAllMarkers function was used to calculate the differentially expressed genes of each subtype with log_2_ fold change = 0.5. A large number of classical marker genes specifically expressed for different cell subtypes were collected. After annotating the main cell subtypes, the cell subtypes were classified and annotated in more detail in the same way, such as T cells can be annotated as CD4^+^ cells, CD8^+^ cells, etc.

To quantify the enrichment of cell subtypes in different tissues, the method reported in the previous study was adopted to calculate the ratio of the observed and expected number of subtype cells in the tissues.^[^ ^36^ ^]^ The formula was as follows:

(1) $$R_{o/e}=\frac{\mathrm{Observed}}{\mathrm{Expected}}$$

The observed value was the actual number of subtype cells in specific tissue, and the expected value was obtained by counting the number of subtype cells and the number of specific tissues using Chi‐square test. When the *R*_*o*/*e*_ > 1, this subtype was considered to be enriched in this tissue.

### Bacteriological Analysis of Murine Pancreatic Tissue

The pancreas of mice was removed under sterile conditions, weighed, and immediately homogenized in sterile PBS. Homogenates were plated on LB Agar plates and cultured for 24 h at 37 °C. Colonies were counted and CFU/mg pancreatic tissue were calculated.

### RNA Interference

siRNAs were transfected into isolated Tregs with Lipofectamine 3000 reagent (Invitrogen) following the manufacturer's instructions. Mouse Ube2k‐specific siRNA was designed and synthesized by Hanheng Biotechnology. The efficiency of interference was determined by qPCR.

### Immunoprecipitation, Pull‐Dwn and Immunoblotting Assays

HEK293T cells were co‐transfected with plasmid as indicated for 48 h and lysed with cell lysis buffer for Western blot and immunoprecipitation (Beyotime). Whole‐cell lysates (WCL) were used as input and were subjected to IP with 2 µg antibody overnight. 50 µL protein G magnetic beads (Bio‐Rad) prewashed with 0.1% PBST were added and incubated for another 4 h at 4 °C. Protein complex‐containing beads were washed four times with 0.1% PBST. Proteins were eluted and boiled in 1% SDS loading buffer.

For pull down assays, Dynabeads His‐tag magnetic beads (Invitrogen) were preincubated with either vehicle or his‐tagged Amuc_1409 protein in PBS for 2 h at room temperature, then washed with 0.01% PBST, and incubated with Tregs cell lysates overnight at 4 °C. The beads were washed four times with 0.01% PBST and analyzed by immunoblot.

Tissues and cells treated as indicated were lysed with RIPA lysis buffer (Beyotime). The lysates were subjected to 12% SDS–polyacrylamide gel electrophoresis and then transferred to polyvinylidene difluoride membranes (Millipore). Proteins were further incubated with the indicated primary antibodies and then horseradish peroxidase (HRP)‐conjugated secondary antibodies. Protein bands were probed using an enhanced chemiluminescence kit (Vazyme) with a ChemiDoc Touch Gel Imaging System (Bio‐Rad).

### Plasmid Constructs

Full‐length cDNAs of mice Pbmb2, Ube2k, Foxp3, ubiquitin, ubiquitin‐K48R, ubiquitin‐K63R fragments were synthesized, then subcloned into pcDNA3.1 vectors (Clontech). All constructs were confirmed by DNA sequencing.

### Statistics

Statistical analyses were performed with Prism GraphPad softwarev.10.1.1. Error bars represented standard errors of the means in all figures, and the two‐sided *p‐*values were examined by one‐way ANOVA with Dunnett's multiple comparisons test or Student's *t‐*test. *R^2^* and exact two‐sided *p‐*values calculated by Pearson's test. A two‐sided *p*‐value < 0.05 was considered statistically significant.

## Conflict of Interest

The authors declare no conflict of interest.

## Author Contributions

J.X., L.D., and Y.L. contributed equally to this study. H.Y., F.G., and J.X. designed the experiments. J.X., L.D., and X. Z. performed the experiments. J.X. and Y.L. conducted bioinformatics analysis. X.G., Y.T., B.S., and X.Y. commented on and revised drafts of the manuscript. J.X. wrote the paper. H.Y., F.G., X.Y., and J.X. supervised research, coordination and strategy.

## Supporting information



Supporting Information

Supporting Information

## Data Availability

The data that support the findings of this study are available from the corresponding author upon reasonable request.
