# Supplementary material for: Akkermansia Muciniphila Alleviates Severe Acute Pancreatitis via Amuc1409‐Ube2k‐Foxp3 Axis in Regulatory T Cells
Source: Adv Sci (Weinh). 2025 Jun 4;12(30):e04214. doi: 10.1002/advs.202504214 (PMC12376690; doi:10.1002/advs.202504214)
Supplement: Supplementary file 1 — Supporting Information [file ADVS-12-e04214-s002.docx]

*Akkermansia muciniphila* Alleviates Severe Acute Pancreatitis via Amuc1409-Ube2k-Foxp3 Axis in Regulatory T Cells

Jinyan Xie^1, 4†^, Lijun Du^2, 4†^, Yunkun Lu^2†^, Xiuliu Guo^1^, Xinyuan Zhou^2^, Yifan Tong^2^, Bo Shen^2, 4^, Xin Yu^3, 4*^, Feng Guo^1, 4*^, Hong Yu^2, 4*^

^1^ Department of Critical Care Medicine, Sir Run Run Shaw Hospital, Zhejiang University School of Medicine, Hangzhou, 310016, P. R. China;

^2^ Department of General Surgery, Sir Run Run Shaw Hospital, Zhejiang University School of Medicine, Hangzhou, 310016, P. R. China;

^3^ Department of Anesthesiology, Sir Run Run Shaw Hospital, Zhejiang University School of Medicine, Hangzhou, 310016, P. R. China;

^4^ Zhejiang Key Laboratory of Precise Diagnosis and Treatment of Abdominal Infection, Sir Run Run Shaw Hospital, School of Medicine, Zhejiang University, Hangzhou, 310016, P. R. China.

†Contributed equally to this study.

*Corresponding author.

Email: Xin Yu, [xinxin_yu@zju.edu.cn](mailto:xinxin_yu@zju.edu.cn)

Feng Guo, [3408002@zju.edu.cn](mailto:3408002@zju.edu.cn)

Hong Yu, [blueyu000@zju.edu.cn](mailto:blueyu000@zju.edu.cn)

**Supplementary Figures**

**
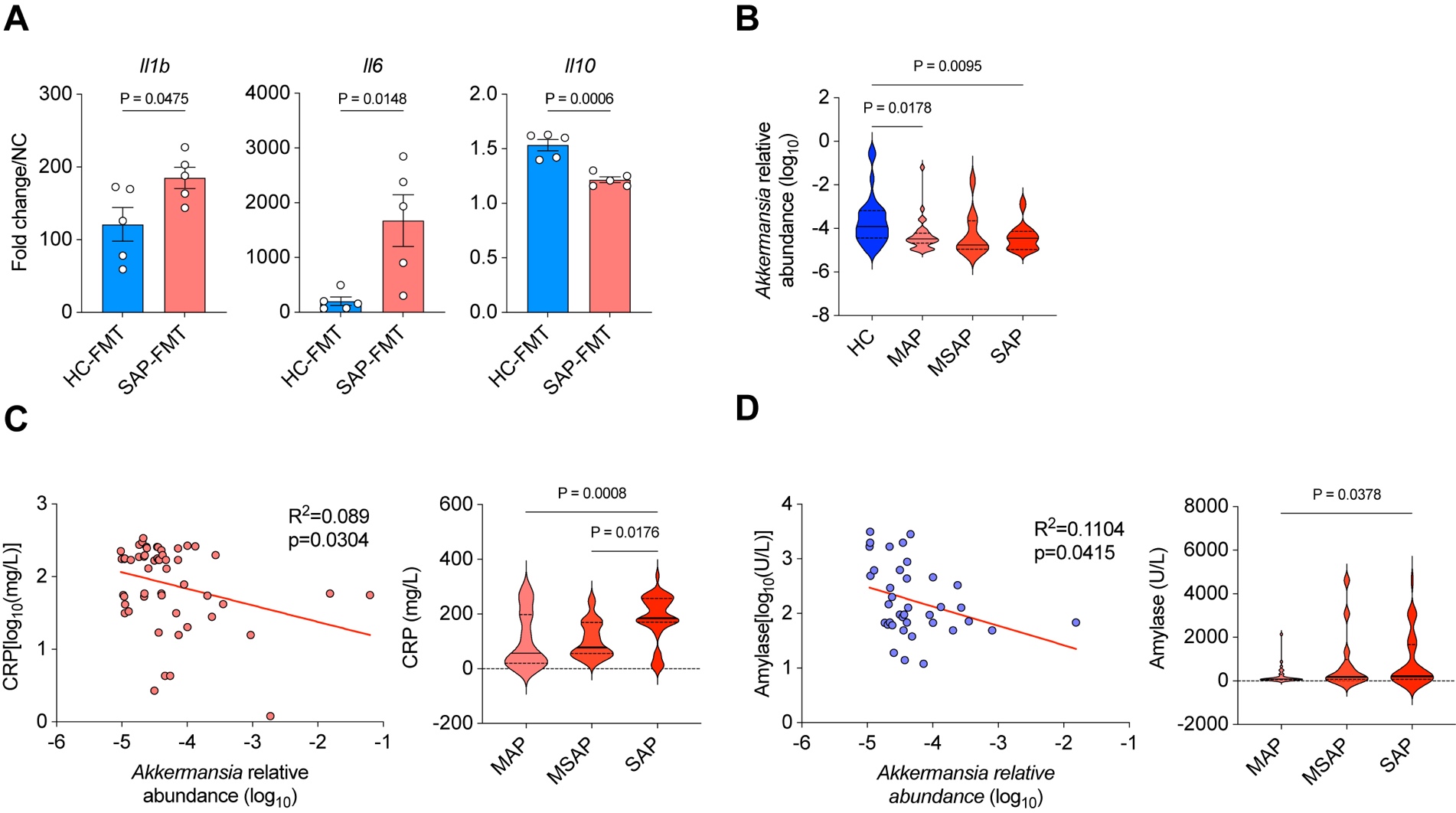
**

**Figure S1. Patients with AP exhibit a decreased fecal abundance of *Akkermansia* that was inversely correlated with the inflammation of AP.** (A) Pancreatic *Il1b, Il6, Il10* mRNA level from Abx-treated mice received fecal microbiota transplantation (FMT) from Healthy control (HC) or patients with SAP donors at 12 hours post caerulein-induced SAP modeling (*n* = 5). (B) Relative abundance of *Akkermansia*. (C-D) the concentrations of CRP (C) and Amylase (D) among the three groups and the correlations with the relative abundance of *Akkermansia*. The two-sided *P* values were examined by Student’s *t* test (A) or one-way ANOVA with Dunnett's multiple comparisons test (B-D) and data were presented as mean ± sem. *R^2^* and exact two-sided *P* values calculated by Pearson’s test are shown (C-D).

**
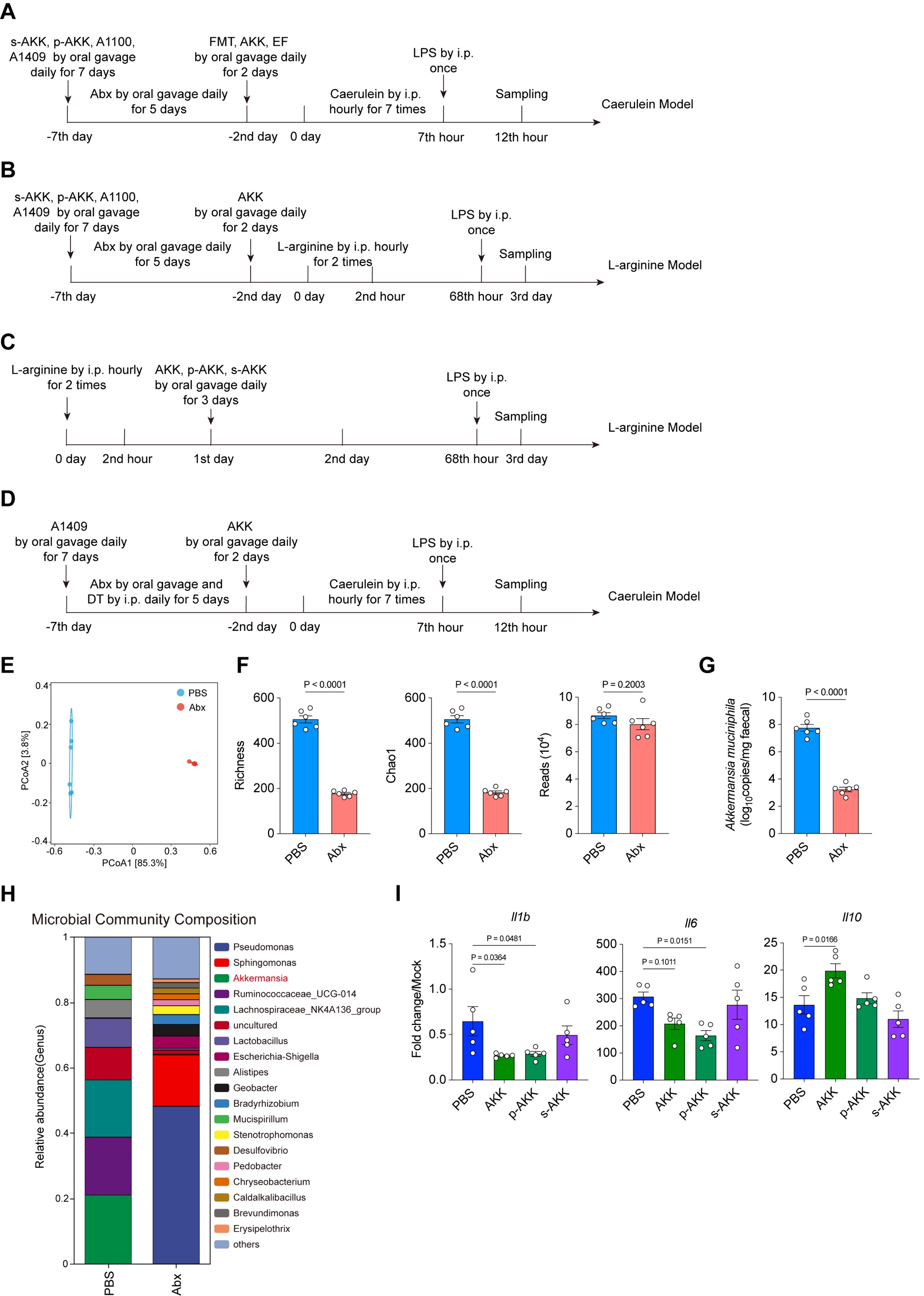
**

**Figure S2. *Akkermansia muciniphila* attenuated pancreatic inflammation.** (A-D) Schematic representation of treatment with Abx, FMT, bacteria, Amuc1100, Amuc1409, and Caerulein or L-arginine constructed SAP model. The 16S rRNA sequencing analysis of fecal samples from PBS-treated or Abx-treated mice. (E) PCoA, (F) Alpha diversity indicators of Richness (left) and Chao (middle), total reads (right). (G) Genomic copies of *A. muciniphila*. (H) The microbial community composition at the genus level (n=6). (I) pancreatic *Il1b, Il6, Il10* mRNA level from L-arginine-induced SAP mice treated with AKK, p-AKK, s-AKK at 3 days post modeling (*n* = 5). The two-sided *P* values were examined by Student’s *t* test (F-G) or one-way ANOVA with Dunnett's multiple comparisons test (I) and data were presented as mean ± sem.

**
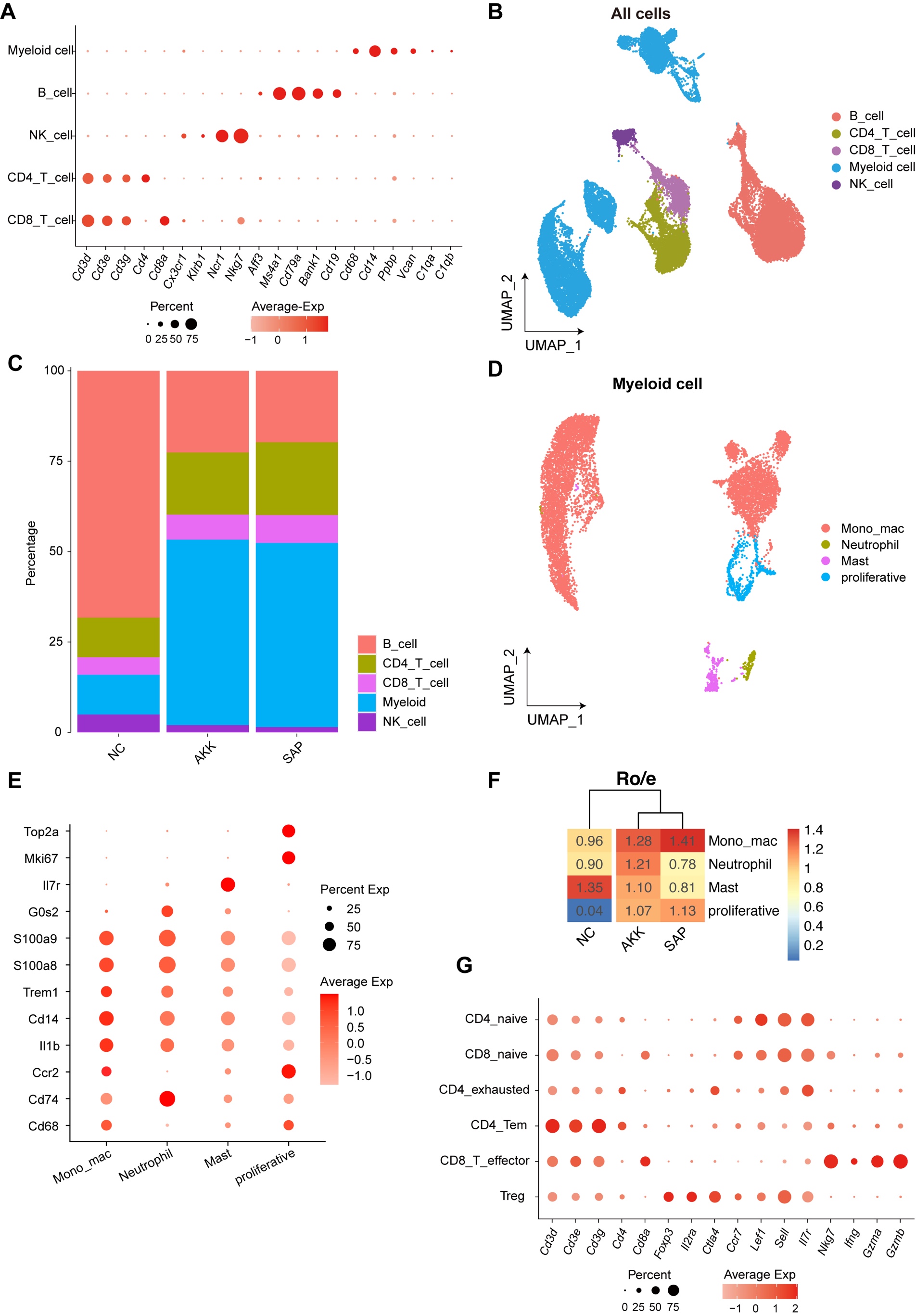
**

**Figure S3. *Akkermansia muciniphila* did not upregulate peripheral macrophages and neutrophils.** L-arginine-induced SAP mice treated with *A. muciniphila* (AKK) daily for 3 days. PBMCs were isolated at 3 days post modeling and (A) Bubble diagram showing the specific genes expressed by individual immune cells. (B) UMAP plots of all immune cells, including 5 major clusters. (C) Bar plots showing the immune cell proportions in PBMCs. (D) UMAP plots of myeloid cells, including 4 major clusters. (E) Bubble diagram showing the specific genes expressed by individual immune cells. (F) Ro/e of T cells. (G) Bubble diagram showing the specific genes expressed by individual T cells.

**
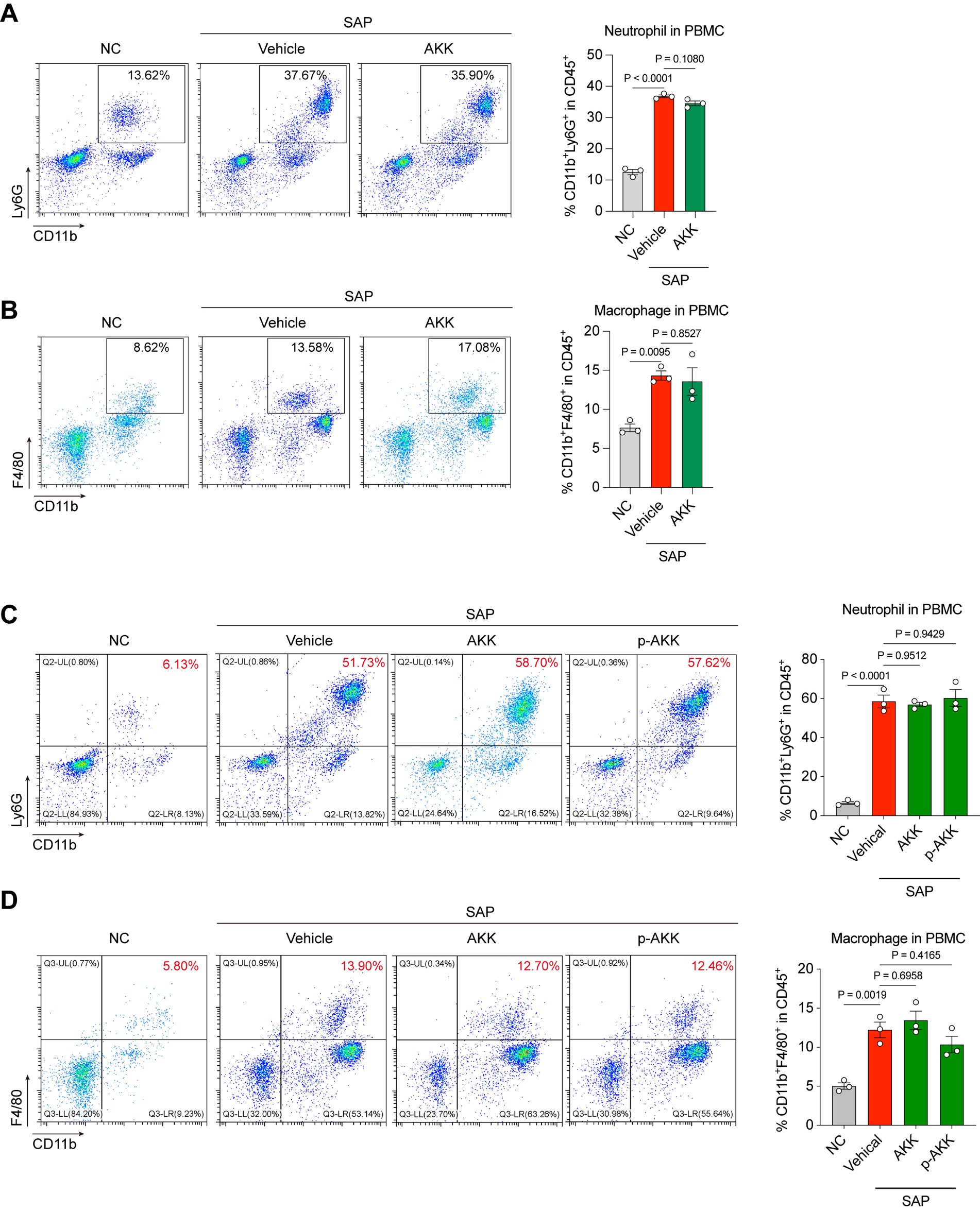
**

**Figure S4. *Akkermansia muciniphila* upregulated peripheral regulatory T cells of PBMCs and spleen.** L-arginine-induced SAP mice treated with *A. muciniphila* (AKK) daily for 3 days. PBMCs were isolated at 3 days post modeling and (A) the percentage of Ly6G^+^/CD11b^+^-cells (*n* = 3), (B) the percentage of F4/80^+^/CD11b^+^-cells were detected by flow cytometry (*n* = 3). Mice treated with Abx or Abx-treated mice colonized with AKK or p-AKK at 12 hours post caerulein-induced SAP modeling. (C) the percentage of Ly6G^+^/CD11b^+^-cells in PBMCs (*n* = 3), (D) the percentage of F4/80^+^/CD11b^+^-cells in PBMCs (n = 3). The two-sided *P* values were examined by one-way ANOVA with Dunnett's multiple comparisons test and data were presented as mean ± sem (A-D).

**
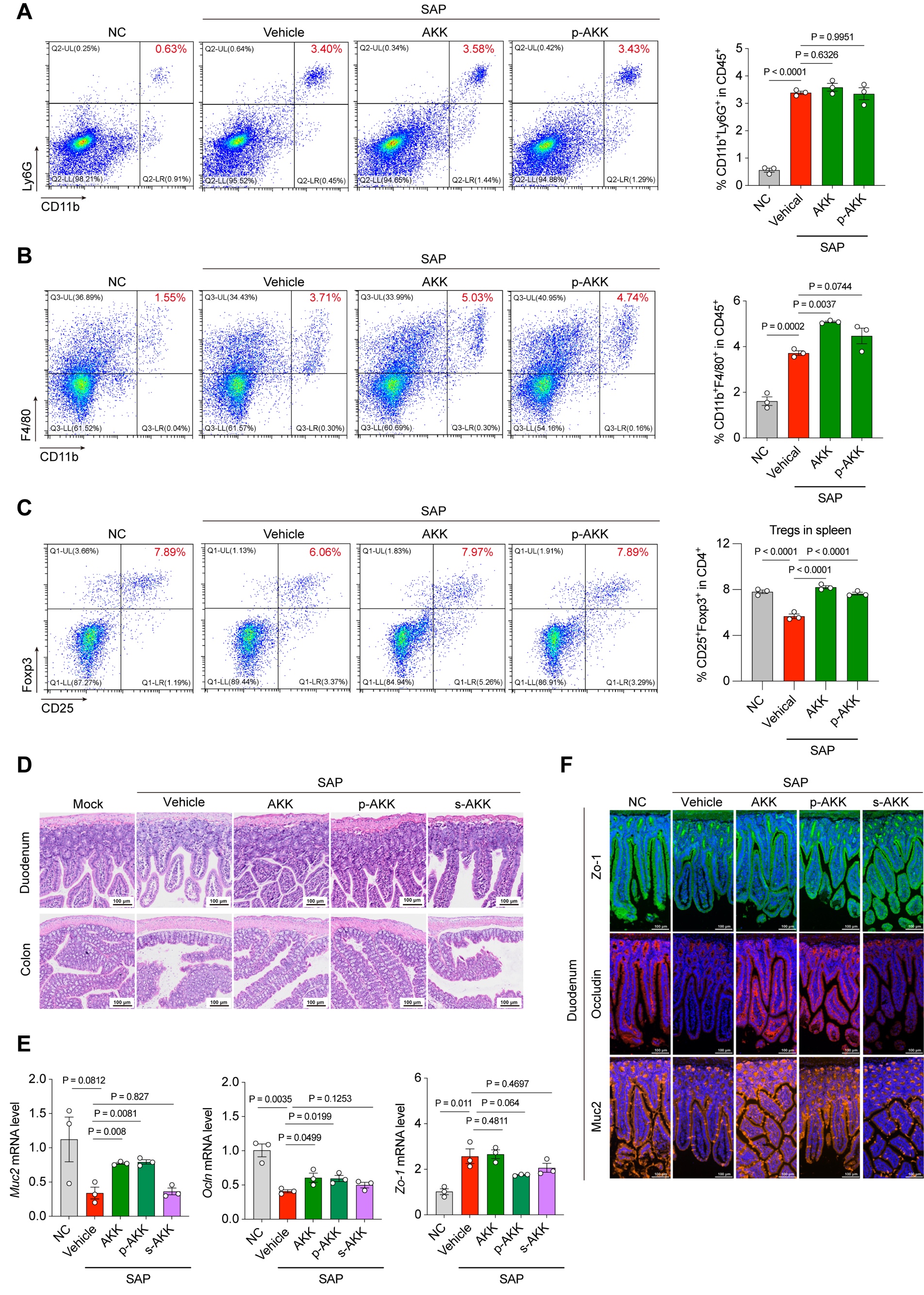
**

**Figure S5. *Akkermansia muciniphila* promotes intestinal barrier integrity.** Mice treated with Abx or Abx-treated mice colonized with AKK or p-AKK at 12 hours post caerulein-induced SAP modeling. (A) the percentage of Ly6G^+^/CD11b^+^-cells in spleen (*n* = 3), (B) the percentage of F4/80^+^/CD11b^+^-cells in spleen (*n* = 3). (C) the percentage of CD4^+^/CD25^+^/Foxp3^+^-Tregs in spleen (*n* = 3). (D) The histopathology of duodenum (upper) and colon (down) was detected by H.E. stain. (E) The mRNA levels of *Muc2*, *Ocln,* and *Zo-1* in the colon (*n* = 3). (F) Representative indirect fluorescent assay (IFA) images of duodenum. Zo-1, Occludin, and Muc2 as makers for tight junction proteins. The two-sided *P* values were examined by one-way ANOVA with Dunnett's multiple comparisons test and data were presented as mean ± sem (A-C, E).

**
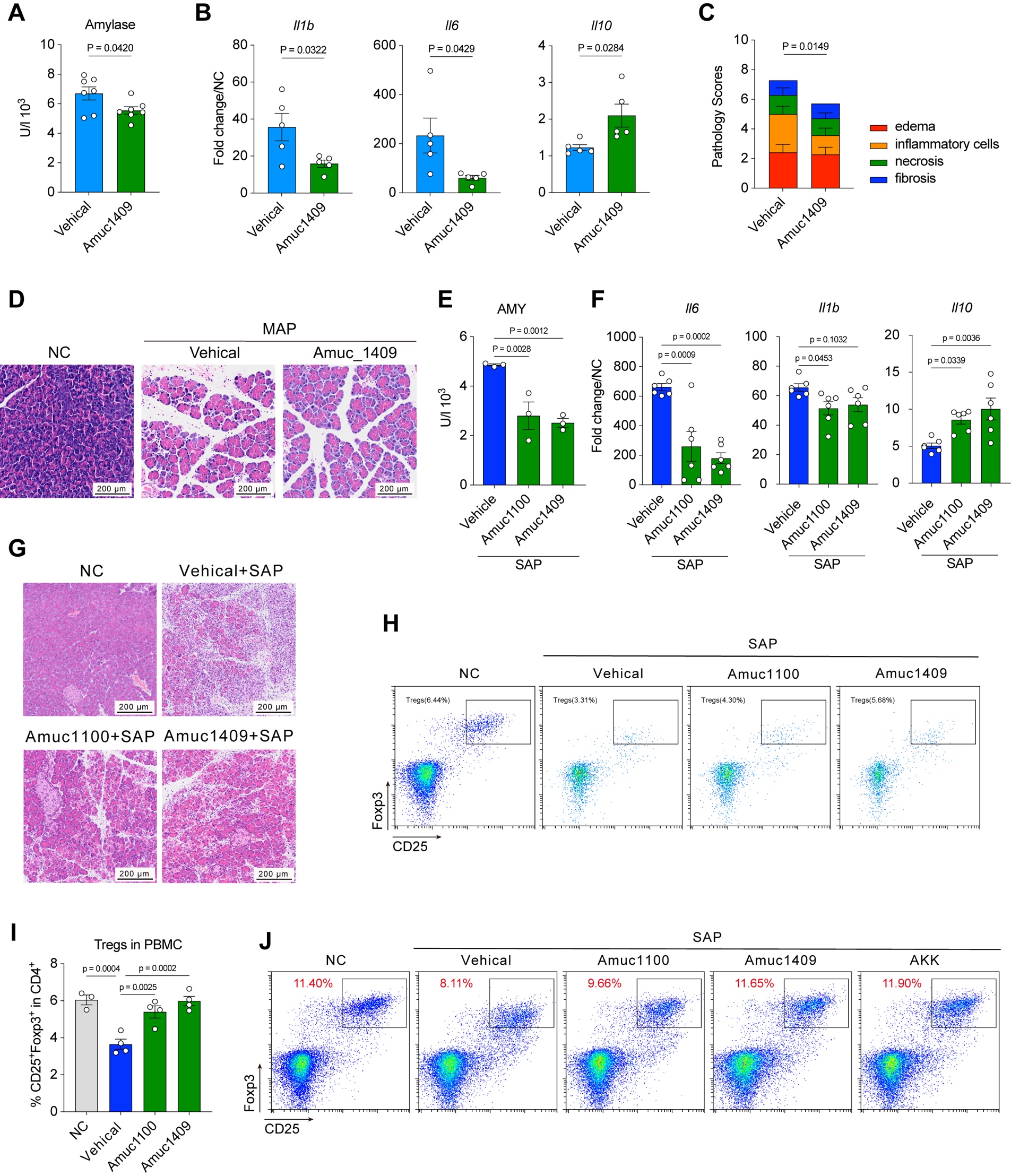
**

**Figure S6. *A. muciniphila*-driven protein Amuc_1409 diminished pancreatic and systemic inflammation**. Mice treated with Abx or Abx-treated mice gavaged with Amuc_1409 at 12 hours post caerulein -induced MAP modeling. (A) Serum amylase level (*n* = 7). (B) pancreatic *Il6*, *Il1b, Il10* mRNA level (*n* = 5). (C) pathology scores, and (D) pancreatic histopathology. Mice treated with Abx or Abx-treated mice gavaged with Amuc_1100 or Amuc_1409 at 72 hours post L-arginine-induced SAP modeling. (E) Serum amylase level (*n* = 3). (F) pancreatic *Il6*, *Il1b, Il10* mRNA level (*n* = 6). (G) pancreatic histopathology. (H) Representative flow cytometry plots, (I) the percentage of CD4^+^/CD25^+^/Foxp3^+^-Tregs in PBMC (*n* = 3-4). (J) Representative flow cytometry plots in MLN. The two-sided *P* values were examined by Student’s *t* test (A-C) or one-way ANOVA with Dunnett's multiple comparisons test (E-F, I) and data were presented as mean ± sem.


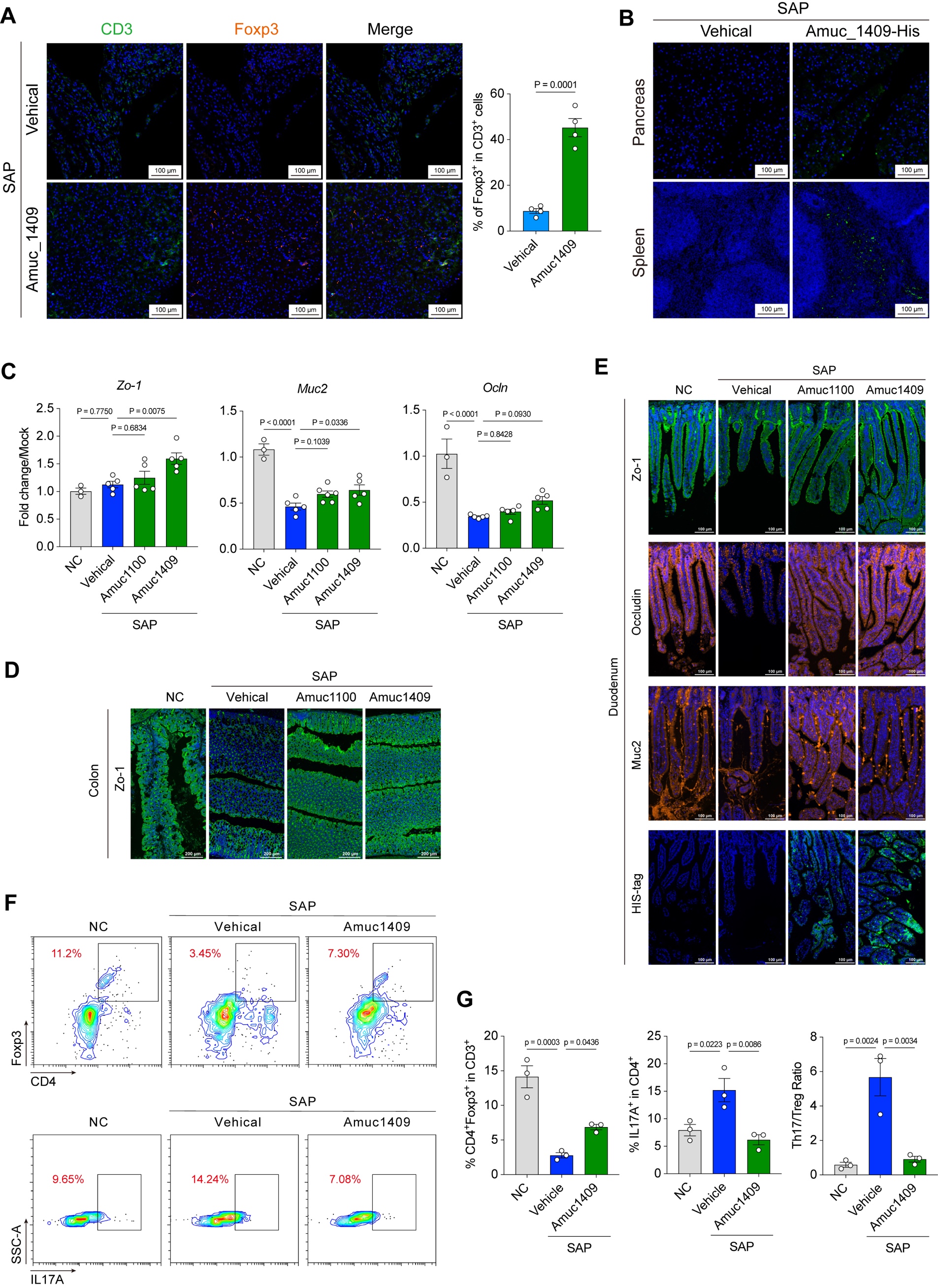


**Figure S7. *A. muciniphila*-driven protein Amuc_1409 ameliorates intestinal barrier dysfunction by resolving Th17/Treg imbalance**. Mice treated with Abx or Abx-treated mice gavaged with Amuc_1100 or Amuc_1409 at 12 hours post caerulein-induced SAP modeling. (A) Representative IFA images of pancreatic CD3 and Foxp3 (right), the percentage of Foxp3^+^ in CD3^+^ cells (right) (*n* = 4). (B) Representative IFA images of pancreas (upper) and spleen (down). His as tag for Amuc_1409. Mice treated with Abx or Abx-treated mice gavaged with Amuc_1100 or Amuc_1409 at 72 hours post L-arginine-induced SAP modeling. (C) colonic *Zo-1*, *Muc2*, and *Ocln* mRNA level (*n* = 3-5). (D-E) Representative IFA images of colon (D) and duodenum (E). Zo-1, Occludin, and Muc2 as makers for tight junction proteins, His as tag for Amuc_1100 and Amuc_1409. (F) Representative flow cytometry plots, (G) the percentage of colonic CD4^+^/Foxp3^+^-Tregs (left), IL17A^+^-Th17 cell (middle), the ratio of Th17/Treg (down) (*n* = 3). The two-sided *P* values were examined by Student’s *t* test (A) or one-way ANOVA with Dunnett's multiple comparisons test (C, G) and data were presented as mean ± sem.


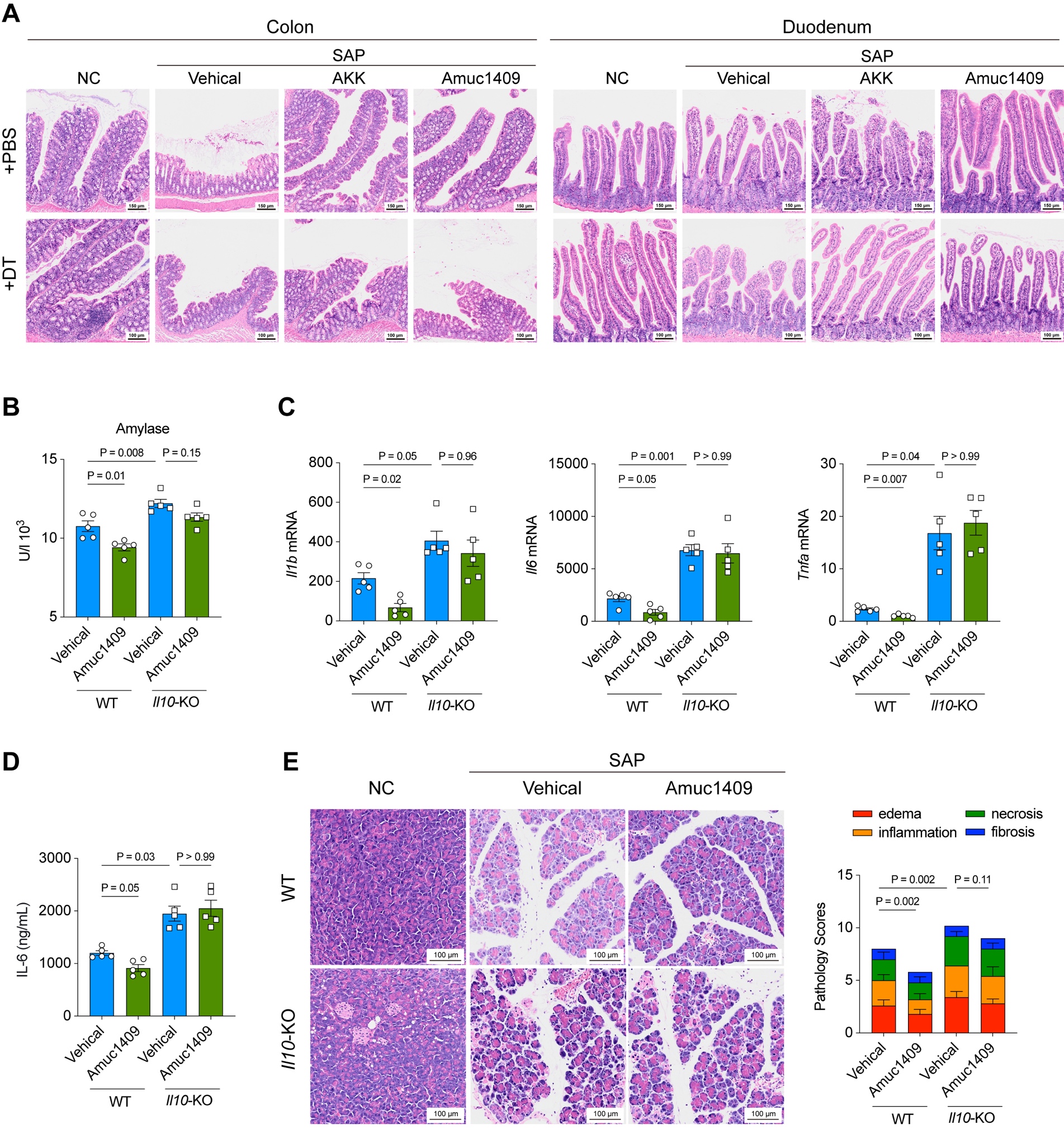


**Figure S8. Amuc_1409 protects the host from SAP in a IL10-dependent manner.** Foxp3-DTR mice were intraperitoneally injected with diphtheria toxin (DT) daily for 5 days, AKK or Amuc_1409 were gavaged to Tregs-depletion mice and induced SAP using caerulein. (A) The histopathology of colon (left) and duodenum (right) was detected by H.E. stain. Amuc_1409 were gavaged to IL-10-KO mice and induced SAP using caerulein (B) Serum amylase level (*n* = 5), (C) pancreatic *Il6*, *Il1b*, *Tnfa* mRNA level (*n* = 5), and (D) serum IL-6 level (*n* = 5), (E) representative pancreatic histopathology (left) and pathology scores (right). The two-sided *P* values were examined by one-way ANOVA with Tukey's multiple comparisons test and data were presented as mean ± sem (B-E).

**
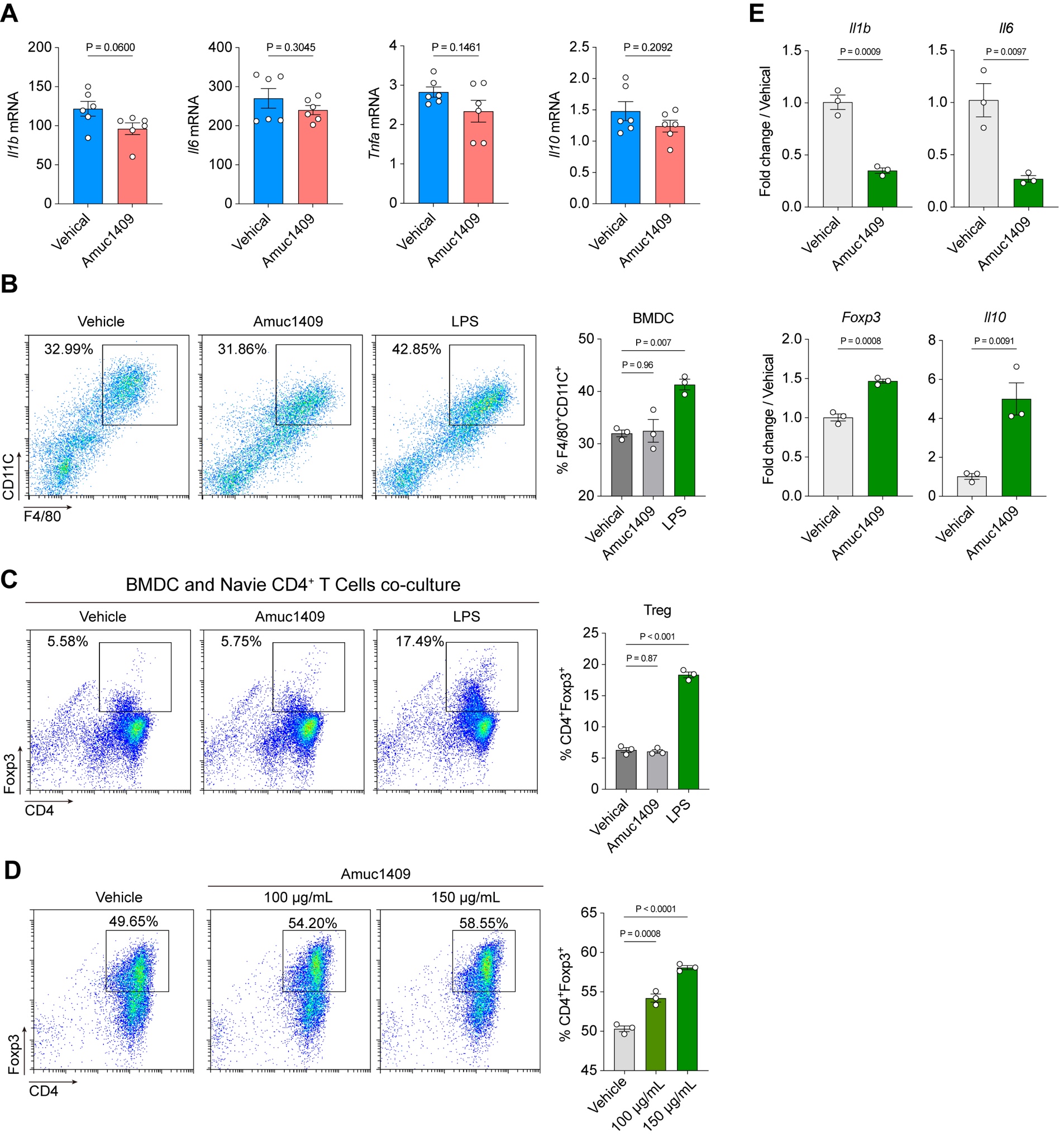
**

**Figure S9. Amuc_1409 promoted Tregs differentiation in a DCs-independent manner.** (A) Murine bone marrow-derived macrophages (BMDMs) were induced in vitro, stimulated with Amuc_1409 (100 μg/mL) or LPS (1 μg/mL), mRNA levels of *Il1b*, *Il6*, *Tnfa* and *Il10* (*n* = 6). Murine bone marrow-derived DCs (BMDCs) were induced in vitro, stimulated with Amuc_1409 (100 μg/mL) or LPS (1 μg/mL), and cocultured with Naïve CD4^+^ T cells to assess Tregs differentiation. (B) Representative flow cytometry plots (left), and the percentage of F4/80^+^/CD11C^+^-cells (right) (n = 3). (C) Representative flow cytometry plots (left), and the percentage of Foxp3^+^/CD4^+^-cells (right) (n = 3). (D-E) Differentiated Tregs were treated with or without different does of Amuc_1409 under IL-2 and TGF-β condition. (D) Representative flow cytometry plots (left), and the percentage of CD4^+^/Foxp3^+^-Tregs (right) (*n* = 3). (E) mRNA levels of *Il1b*, *Il6*, *Foxp3* and *Il10* (*n* = 3). The two-sided *P* values were examined by Student’s *t* test (A, E) or one-way ANOVA with Dunnett's multiple comparisons test (B-D) and data were presented as mean ± sem.

**
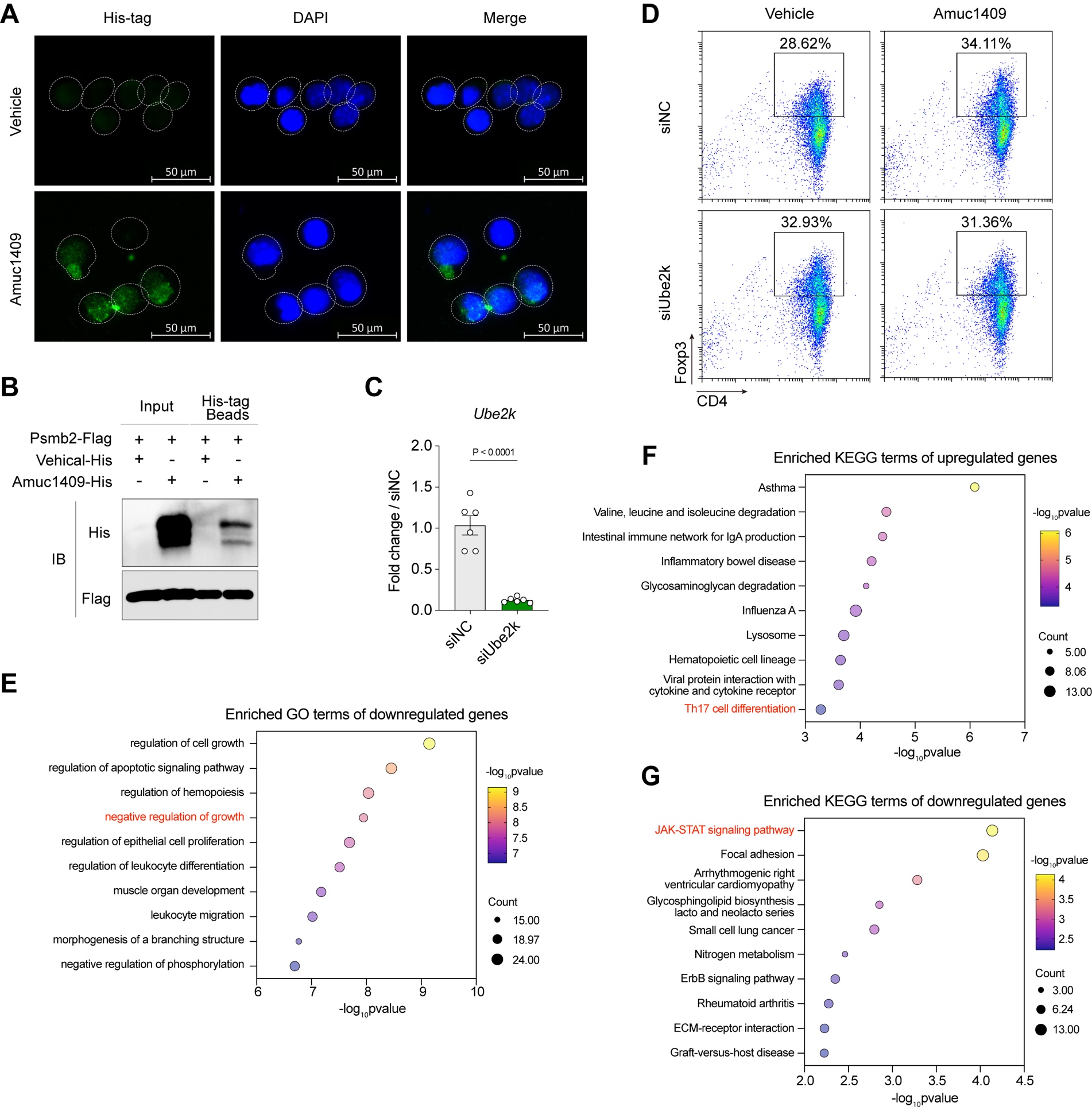
**

**Figure S10. Amuc_1409 promoted Tregs differentiation through interaction with Ube2k.** (A) Representative IFA images of Amuc_1409 in Tregs. (B) HEK293T cells were co-transfected with pcDNA3.1-Amuc_1409-His and pcDNA3.1-Psmb2-Flag. Co-Immunoprecipitation assay was performed at 48 hours-post transfections. Representative Western blot. (C-D) Differentiated Tregs were transfected with siNC or siUbe2k, and treated with or without 200 μg/mL of Amuc_1409 under IL-2 and TGF-β condition. (C) mRNA level of Ube2k (*n* = 6). (D) Representative flow cytometry plots. (E-G) Transcriptome analysis of differentiated Tregs transfected with siNC or siUbe2k. (E) Bubble plots of GO enrichment in downregulated genes, and (F-G) KEGG pathways enriched in upregulated (F) and downregulated (G) genes in siUbe2k versus siNC transfected Tregs. Cycle size represented the gene numbers in each pathway, color gradients represented the adjusted P value. The two-sided *P* values were examined by Student’s *t* test (C) and data were presented as mean ± sem.

**Reference**

[1] M. A. Mederos, H. A. Reber, M. D. Girgis, *Jama* **2021**, *325* (4), 382, <https://doi.org/10.1001/jama.2020.20317>.

[2] a) P. K. Garg, V. P. Singh, *Gastroenterology* **2019**, *156* (7), 2008, <https://doi.org/10.1053/j.gastro.2018.12.041>; b) J. A. Greenberg, J. Hsu, M. Bawazeer, J. Marshall, J. O. Friedrich, A. Nathens, N. Coburn, G. R. May, E. Pearsall, R. S. McLeod, *Can J Surg* **2016**, *59* (2), 128, <https://doi.org/10.1503/cjs.015015>.

[3] a) W. Halangk, M. M. Lerch, B. Brandt-Nedelev, W. Roth, M. Ruthenbuerger, T. Reinheckel, W. Domschke, H. Lippert, C. Peters, J. Deussing, *J Clin Invest* **2000**, *106* (6), 773, <https://doi.org/10.1172/jci9411>; b) L. Zierke, D. John, M. Gischke, Q. T. Tran, M. Sendler, F. U. Weiss, U. T. Bornscheuer, C. Ritter, M. M. Lerch, A. A. Aghdassi, *Cell Mol Life Sci* **2024**, *81* (1), 207, <https://doi.org/10.1007/s00018-024-05247-7>.

[4] A. S. Gukovskaya, I. Gukovsky, H. Algül, A. Habtezion, *Gastroenterology* **2017**, *153* (5), 1212, <https://doi.org/10.1053/j.gastro.2017.08.071>.

[5] a) X. Liu, W. Luo, J. Chen, C. Hu, R. N. Mutsinze, X. Wang, Y. Zhang, L. Huang, W. Zuo, G. Liang, Y. Wang, *Cell Mol Gastroenterol Hepatol* **2022**, *14* (5), 1103, <https://doi.org/10.1016/j.jcmgh.2022.07.013>; b) P. J. Lee, G. I. Papachristou, *Nat Rev Gastroenterol Hepatol* **2019**, *16* (8), 479, <https://doi.org/10.1038/s41575-019-0158-2>.

[6] M. Sendler, C. van den Brandt, J. Glaubitz, A. Wilden, J. Golchert, F. U. Weiss, G. Homuth, L. L. De Freitas Chama, N. Mishra, U. M. Mahajan, L. Bossaller, U. Völker, B. M. Bröker, J. Mayerle, M. M. Lerch, *Gastroenterology* **2020**, *158* (1), 253, <https://doi.org/10.1053/j.gastro.2019.09.040>.

[7] R. S. Hotchkiss, G. Monneret, D. Payen, *Nat Rev Immunol* **2013**, *13* (12), 862, <https://doi.org/10.1038/nri3552>.

[8] J. Liu, L. Huang, M. Luo, X. Xia, *Crit Rev Microbiol* **2019**, *45* (5-6), 539, <https://doi.org/10.1080/1040841x.2019.1621795>.

[9] H. Li, J. Xie, X. Guo, G. Yang, B. Cai, J. Liu, M. Yue, Y. Tang, G. Wang, S. Chen, J. Guo, X. Qi, D. Wang, H. Zheng, W. Liu, H. Yu, C. Wang, S. J. Zhu, F. Guo, *Gut Microbes* **2022**, *14* (1), 2127456, <https://doi.org/10.1080/19490976.2022.2127456>.

[10] a) L. J. Wang, Y. L. Jin, W. L. Pei, J. C. Li, R. L. Zhang, J. J. Wang, W. Lin, *Acta Pharmacol Sin* **2024**, *45* (3), 570, <https://doi.org/10.1038/s41401-023-01186-4>; b) G. Li, L. Liu, T. Lu, Y. Sui, C. Zhang, Y. Wang, T. Zhang, Y. Xie, P. Xiao, Z. Zhao, C. Cheng, J. Hu, H. Chen, D. Xue, H. Chen, G. Wang, R. Kong, H. Tan, X. Bai, Z. Li, F. McAllister, L. Li, B. Sun, *Nature communications* **2023**, *14* (1), 6179, <https://doi.org/10.1038/s41467-023-41950-y>.

[11] M. Qi-Xiang, F. Yang, H. Ze-Hua, Y. Nuo-Ming, W. Rui-Long, X. Bin-Qiang, F. Jun-Jie, H. Chun-Lan, Z. Yue, *Gut Microbes* **2022**, *14* (1), 2112882, <https://doi.org/10.1080/19490976.2022.2112882>.

[12] E. J. Kang, J. H. Kim, Y. E. Kim, H. Lee, K. B. Jung, D. H. Chang, Y. Lee, S. Park, E. Y. Lee, E. J. Lee, H. B. Kang, M. Y. Rhyoo, S. Seo, S. Park, Y. Huh, J. Go, J. H. Choi, Y. K. Choi, I. B. Lee, D. H. Choi, Y. J. Seo, J. R. Noh, K. S. Kim, J. H. Hwang, J. S. Jeong, H. J. Kwon, H. M. Yoo, M. Y. Son, Y. G. Kim, D. H. Lee, T. Y. Kim, H. J. Kwon, M. H. Kim, B. C. Kim, Y. H. Kim, D. Kang, C. H. Lee, *Nature communications* **2024**, *15* (1), 2983, <https://doi.org/10.1038/s41467-024-47275-8>.

[13] L. Wang, L. Tang, Y. Feng, S. Zhao, M. Han, C. Zhang, G. Yuan, J. Zhu, S. Cao, Q. Wu, L. Li, Z. Zhang, *Gut* **2020**, <https://doi.org/10.1136/gutjnl-2019-320105>.

[14] a) D. Esterházy, M. C. C. Canesso, L. Mesin, P. A. Muller, T. B. R. de Castro, A. Lockhart, M. ElJalby, A. M. C. Faria, D. Mucida, *Nature* **2019**, *569* (7754), 126, <https://doi.org/10.1038/s41586-019-1125-3>; b) C. M. Sun, J. A. Hall, R. B. Blank, N. Bouladoux, M. Oukka, J. R. Mora, Y. Belkaid, *J Exp Med* **2007**, *204* (8), 1775, <https://doi.org/10.1084/jem.20070602>.

[15] K. Nutsch, J. N. Chai, T. L. Ai, E. Russler-Germain, T. Feehley, C. R. Nagler, C. S. Hsieh, *Cell Rep* **2016**, *17* (1), 206, <https://doi.org/10.1016/j.celrep.2016.08.092>.

[16] J. Guo, Z. Li, D. Tang, J. Zhang, *Medicine (Baltimore)* **2020**, *99* (31), e21491, <https://doi.org/10.1097/md.0000000000021491>.

[17] a) M. O. Simovic, M. J. Bonham, F. M. Abu-Zidan, J. A. Windsor, *Crit Care Med* **1999**, *27* (12), 2662, <https://doi.org/10.1097/00003246-199912000-00009>; b) E. Fisic, G. Poropat, L. Bilic-Zulle, V. Licul, S. Milic, D. Stimac, *Gastroenterol Res Pract* **2013**, *2013*, 282645, <https://doi.org/10.1155/2013/282645>.

[18] a) X. C. Han, Y. C. Zhang, Y. Wang, M. K. Jia, *Hepatobiliary Pancreat Dis Int* **2003**, *2* (1), 135; b) R. Pezzilli, P. Billi, R. Miniero, B. Barakat, *Dig Dis Sci* **1997**, *42* (7), 1469, <https://doi.org/10.1023/a:1018814710291>.

[19] M. A. Nakasone, K. A. Majorek, M. Gabrielsen, G. J. Sibbet, B. O. Smith, D. T. Huang, *Nat Chem Biol* **2022**, *18* (4), 422, <https://doi.org/10.1038/s41589-021-00952-x>.

[20] Z. Liu, D. S. Lee, Y. Liang, Y. Zheng, J. R. Dixon, *Nature communications* **2023**, *14* (1), 6943, <https://doi.org/10.1038/s41467-023-42647-y>.

[21] X. Ni, W. Kou, J. Gu, P. Wei, X. Wu, H. Peng, J. Tao, W. Yan, X. Yang, A. Lebid, B. V. Park, Z. Chen, Y. Tian, J. Fu, S. Newman, X. Wang, H. Shen, B. Li, B. R. Blazar, X. Wang, J. Barbi, F. Pan, L. Lu, *Embo j* **2019**, *38* (9), <https://doi.org/10.15252/embj.201899766>.

[22] J. Xie, H. Li, X. Zhang, T. Yang, M. Yue, Y. Zhang, S. Chen, N. Cui, C. Yuan, J. Li, S. J. Zhu, W. Liu, *Nature microbiology* **2023**, *8* (1), 91, <https://doi.org/10.1038/s41564-022-01279-6>.

[23] S. M. Kim, S. Park, S. H. Hwang, E. Y. Lee, J. H. Kim, G. S. Lee, G. Lee, D. H. Chang, J. G. Lee, J. Hwang, Y. Lee, M. Kyung, E. K. Kim, J. H. Kim, T. H. Kim, J. H. Moon, B. C. Kim, G. Ko, S. Y. Kim, J. H. Ryu, J. S. Lee, C. H. Lee, J. Y. Kim, S. Kim, W. J. Lee, M. H. Kim, *Cell host & microbe* **2023**, *31* (6), 1021, <https://doi.org/10.1016/j.chom.2023.05.007>.

[24] a) Q. Zhai, S. Feng, N. Arjan, W. Chen, *Crit Rev Food Sci Nutr* **2019**, *59* (19), 3227, <https://doi.org/10.1080/10408398.2018.1517725>; b) T. Zhang, Q. Li, L. Cheng, H. Buch, F. Zhang, *Microb Biotechnol* **2019**, *12* (6), 1109, <https://doi.org/10.1111/1751-7915.13410>.

[25] C. Schwechheimer, M. J. Kuehn, *Nat Rev Microbiol* **2015**, *13* (10), 605, <https://doi.org/10.1038/nrmicro3525>.

[26] J. Glaubitz, A. Wilden, F. Frost, S. Ameling, G. Homuth, H. Mazloum, M. C. Rühlemann, C. Bang, A. A. Aghdassi, C. Budde, T. Pickartz, A. Franke, B. M. Bröker, U. Voelker, J. Mayerle, M. M. Lerch, F. U. Weiss, M. Sendler, *Gut* **2023**, *72* (7), 1355, <https://doi.org/10.1136/gutjnl-2022-327448>.

[27] a) T. Korn, M. Hiltensperger, *Cytokine* **2021**, *146*, 155654, <https://doi.org/10.1016/j.cyto.2021.155654>; b) M. Nishihara, H. Ogura, N. Ueda, M. Tsuruoka, C. Kitabayashi, F. Tsuji, H. Aono, K. Ishihara, E. Huseby, U. A. Betz, M. Murakami, T. Hirano, *Int Immunol* **2007**, *19* (6), 695, <https://doi.org/10.1093/intimm/dxm045>.

[28] A. Fatima, D. Irmak, A. Noormohammadi, M. M. Rinschen, A. Das, O. Leidecker, C. Schindler, V. Sánchez-Gaya, P. Wagle, W. Pokrzywa, T. Hoppe, A. Rada-Iglesias, D. Vilchez, *Commun Biol* **2020**, *3* (1), 262, <https://doi.org/10.1038/s42003-020-0984-3>.

[29] X. Lei, X. Hu, Q. Lu, Y. Yao, W. Sun, Q. Ma, D. Huang, Q. Xu, *Biochem Biophys Res Commun* **2023**, *638*, 210, <https://doi.org/10.1016/j.bbrc.2022.11.046>.

[30] T. Magoč, S. L. Salzberg, *Bioinformatics* **2011**, *27* (21), 2957, <https://doi.org/10.1093/bioinformatics/btr507>.

[31] J. G. Caporaso, J. Kuczynski, J. Stombaugh, K. Bittinger, F. D. Bushman, E. K. Costello, N. Fierer, A. G. Peña, J. K. Goodrich, J. I. Gordon, G. A. Huttley, S. T. Kelley, D. Knights, J. E. Koenig, R. E. Ley, C. A. Lozupone, D. McDonald, B. D. Muegge, M. Pirrung, J. Reeder, J. R. Sevinsky, P. J. Turnbaugh, W. A. Walters, J. Widmann, T. Yatsunenko, J. Zaneveld, R. Knight, *Nat Methods* **2010**, *7* (5), 335, <https://doi.org/10.1038/nmeth.f.303>.

[32] a) M. Sauter, R. J. Sauter, H. Nording, M. Olbrich, F. Emschermann, H. F. Langer, *STAR Protoc* **2022**, *3* (3), 101664, <https://doi.org/10.1016/j.xpro.2022.101664>; b) Y. Mao, D. Shi, G. Li, P. Jiang, *Mol Cell* **2022**, *82* (3), 527, <https://doi.org/10.1016/j.molcel.2021.12.006>.

[33] X. L. Yang, G. Wang, J. Y. Xie, H. Li, S. X. Chen, W. Liu, S. J. Zhu, *mBio* **2021**, *12* (3), <https://doi.org/10.1128/mBio.00366-21>.

[34] Q. Zhang, J. Hu, J. W. Feng, X. T. Hu, T. Wang, W. X. Gong, K. Huang, Y. X. Guo, Z. Zou, X. Lin, R. Zhou, Y. Q. Yuan, A. D. Zhang, H. Wei, G. Cao, C. Liu, L. L. Chen, M. L. Jin, *Genome Biol* **2020**, *21* (1), 99, <https://doi.org/10.1186/s13059-020-02007-1>.

[35] S. Chen, Y. Zhou, Y. Chen, J. Gu, *Bioinformatics* **2018**, *34* (17), i884, <https://doi.org/10.1093/bioinformatics/bty560>.

[36] X. Guo, Y. Zhang, L. Zheng, C. Zheng, J. Song, Q. Zhang, B. Kang, Z. Liu, L. Jin, R. Xing, R. Gao, L. Zhang, M. Dong, X. Hu, X. Ren, D. Kirchhoff, H. G. Roider, T. Yan, Z. Zhang, *Nat Med* **2018**, *24* (7), 978, <https://doi.org/10.1038/s41591-018-0045-3>.
