# Supplementary material for: Akkermansia Muciniphila Alleviates Severe Acute Pancreatitis via Amuc1409‐Ube2k‐Foxp3 Axis in Regulatory T Cells
Source: Adv Sci (Weinh). 2025 Jun 4;12(30):e04214. doi: 10.1002/advs.202504214 (PMC12376690; doi:10.1002/advs.202504214)

ADVANCED SCIENCE
Open Access

Supporting Information

for *Adv. Sci.*, DOI 10.1002/advs.202504214

Akkermansia Muciniphila Alleviates Severe Acute Pancreatitis via Amuc1409-Ube2k-Foxp3 Axis in Regulatory T Cells

Jinyan Xie, Lijun Du, Yunkun Lu, Xiuliu Guo, Xinyuan Zhou, Yifan Tong, Bo Shen, Xin Yu, Feng Guo* and Hong Yu**

Unprocessed images of gels and western blots

Figure 3I

1、 Ocludin

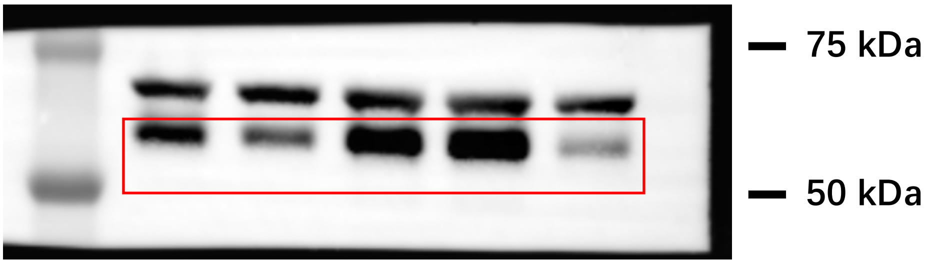

2、 Muc2

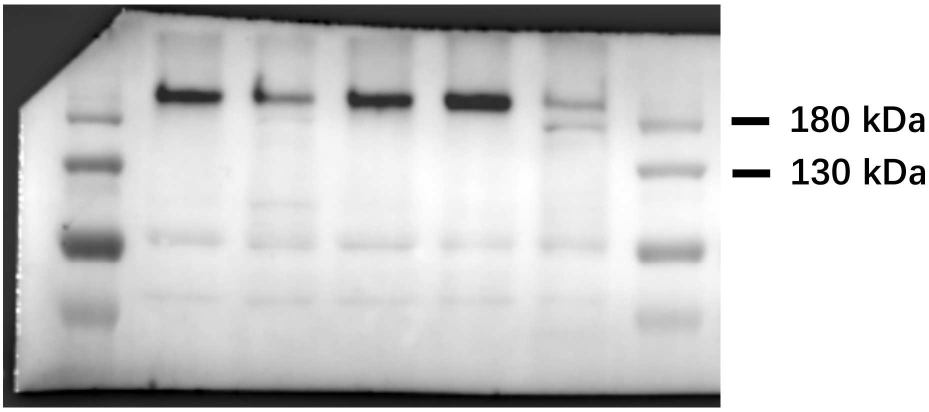

3、 Actin

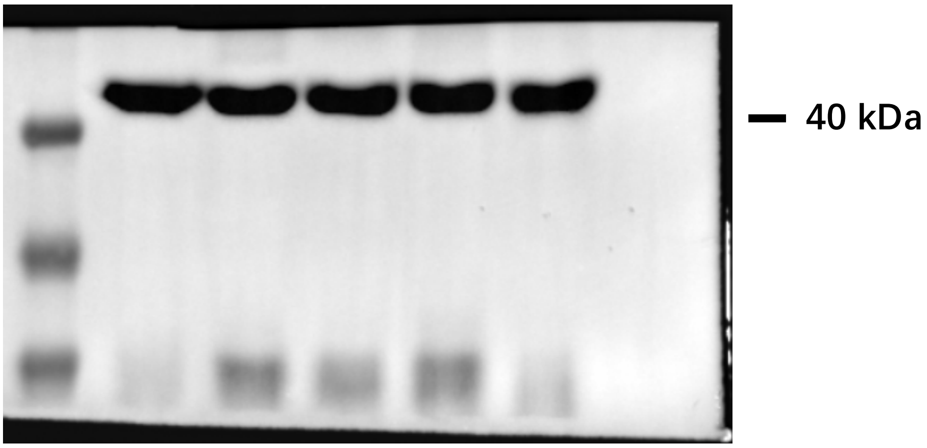

Figure 4I

1、 Ocludin

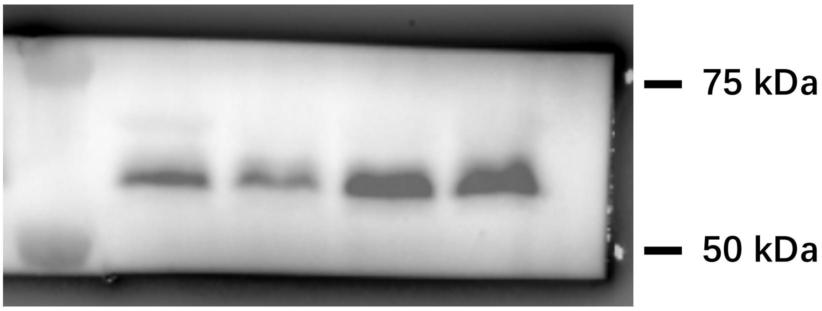

2、 Muc2

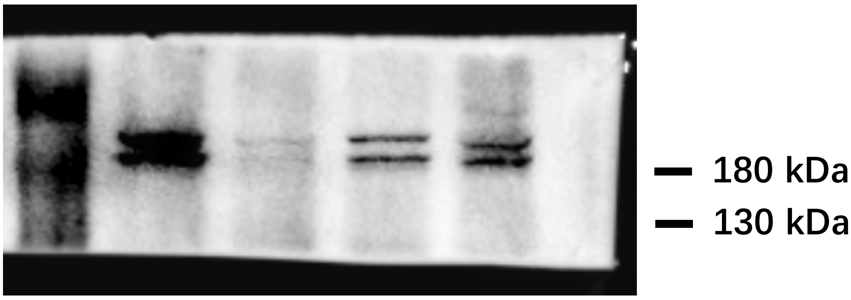

3、 Actin

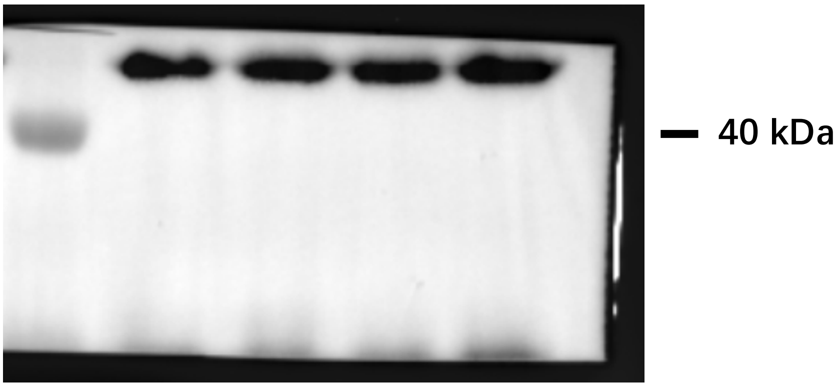

Figure 6C

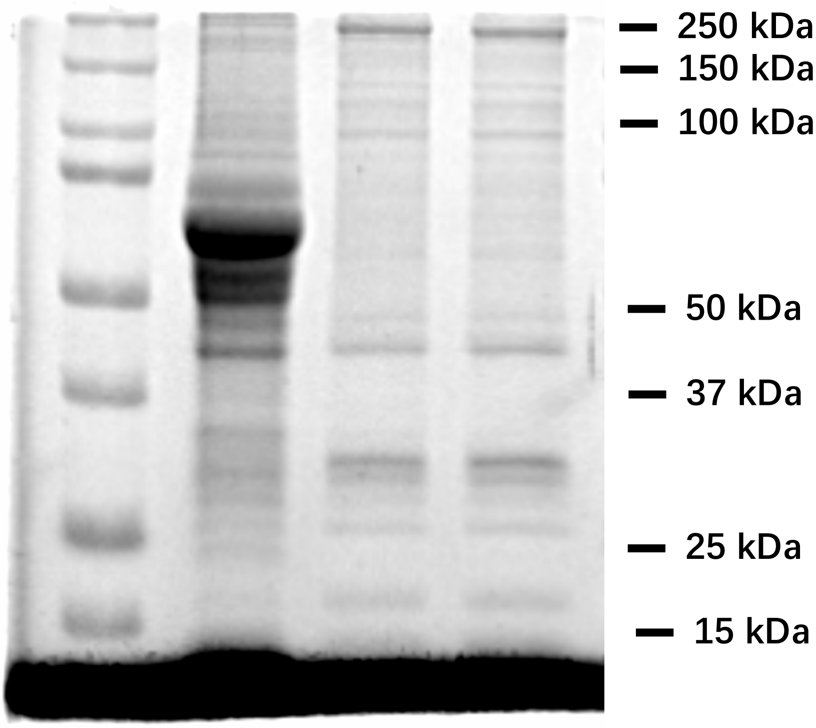

Figure 6D

1、 His

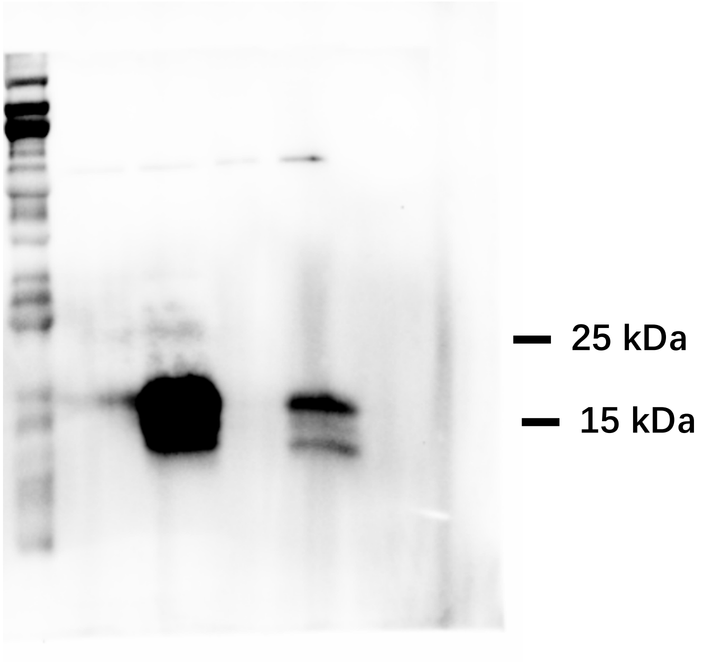

2、 Flag

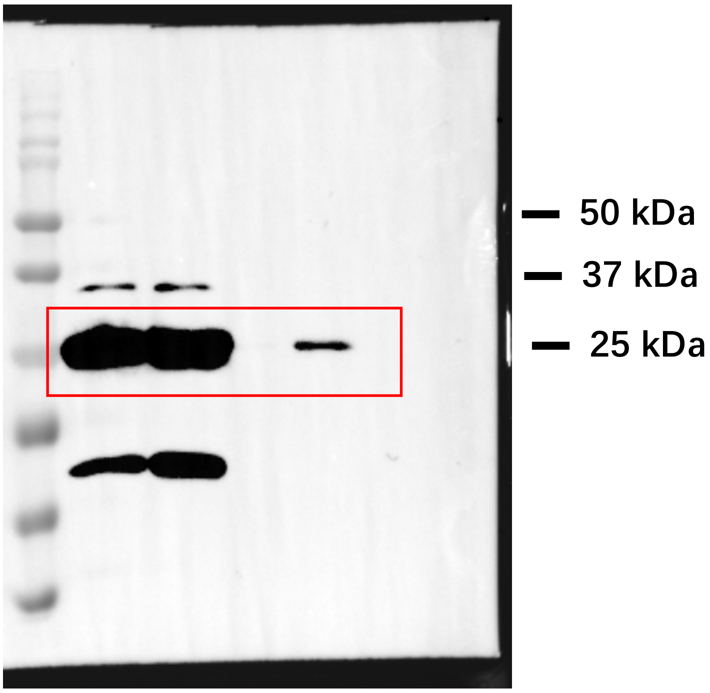

Figure S10B

1、 His

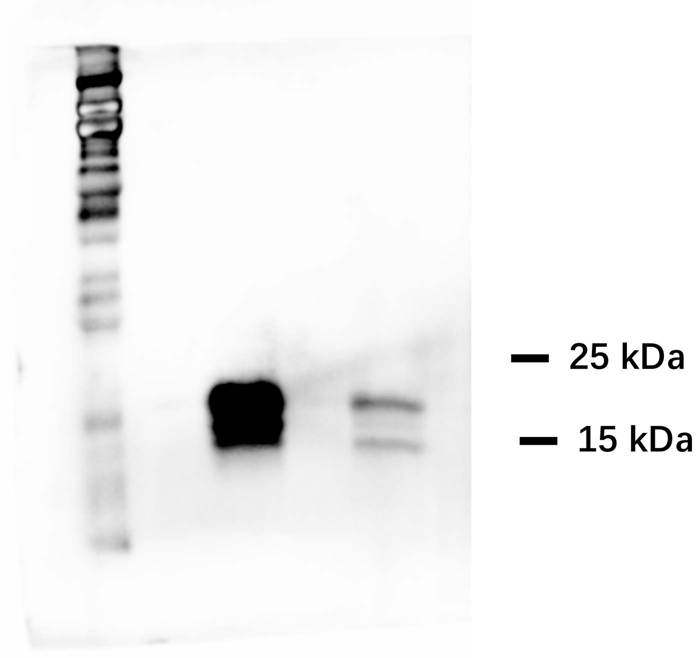

2、 Flag

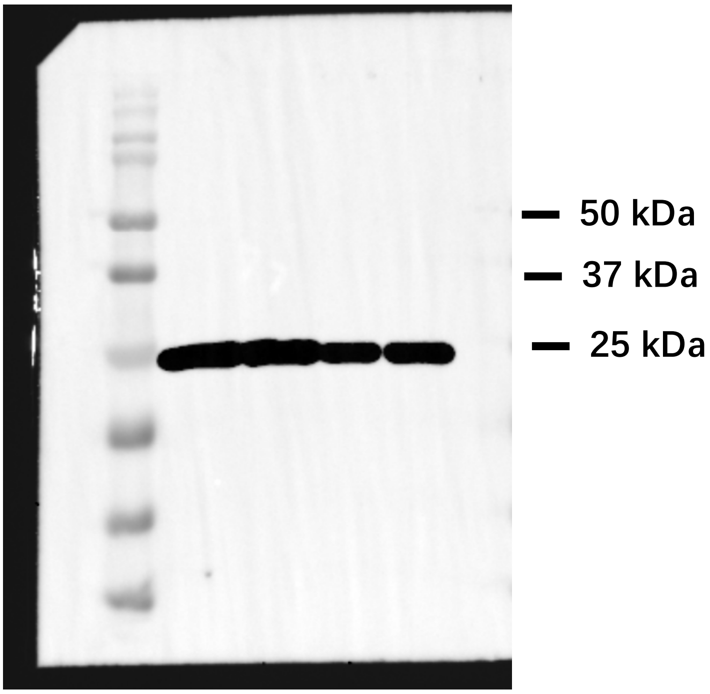

Figure 6I

1、 HA

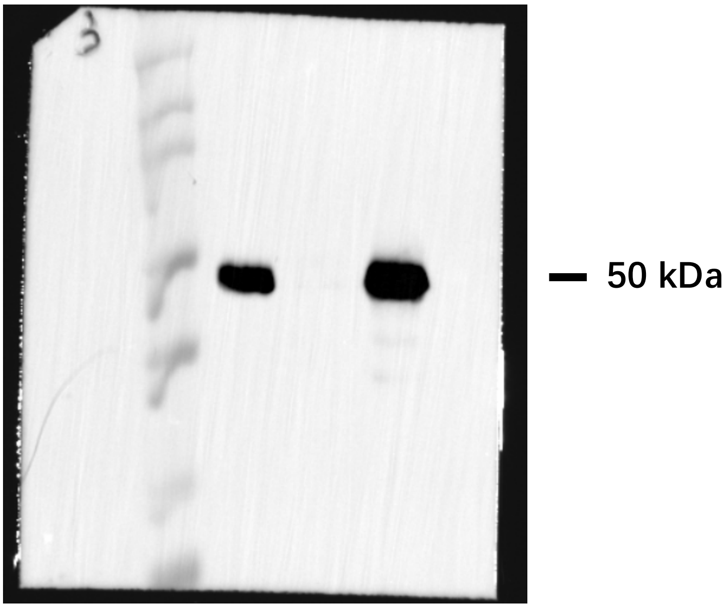

2、 Flag

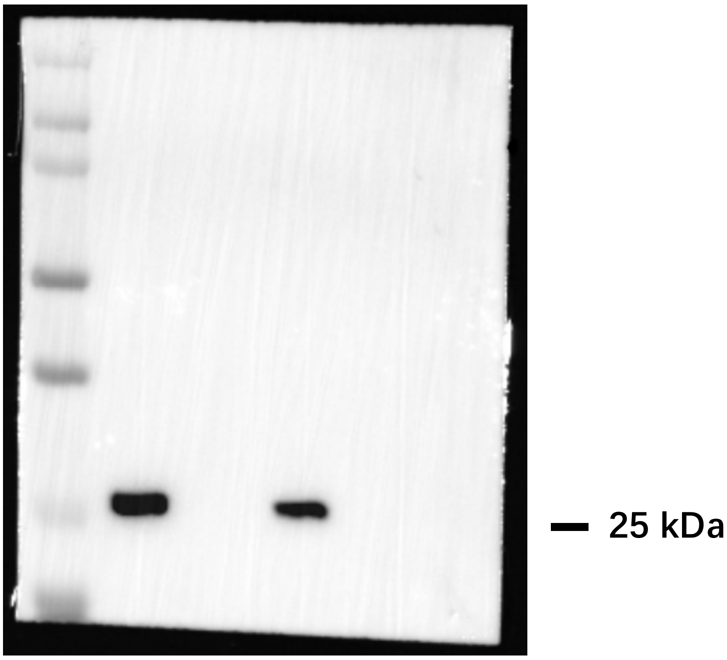

Figure 6J

1、 HA-Input

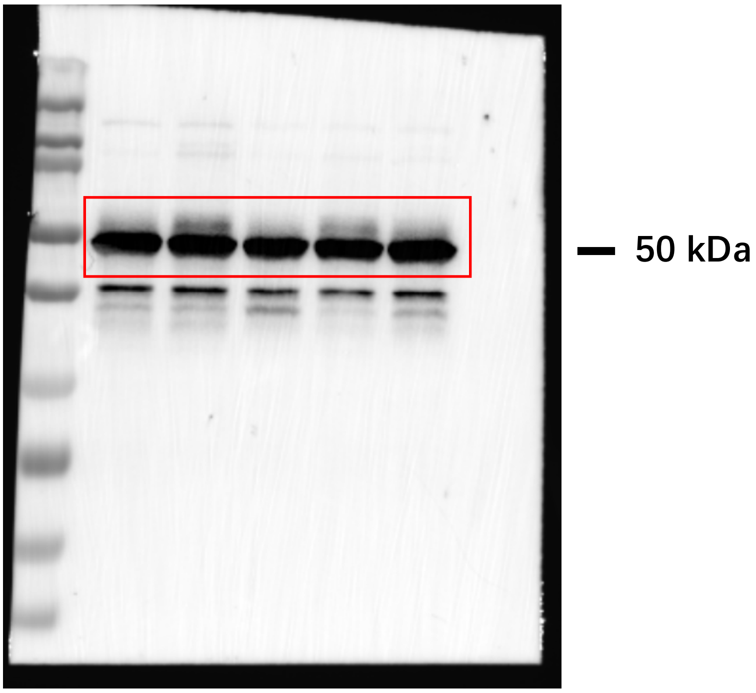

2、 Flag-Input

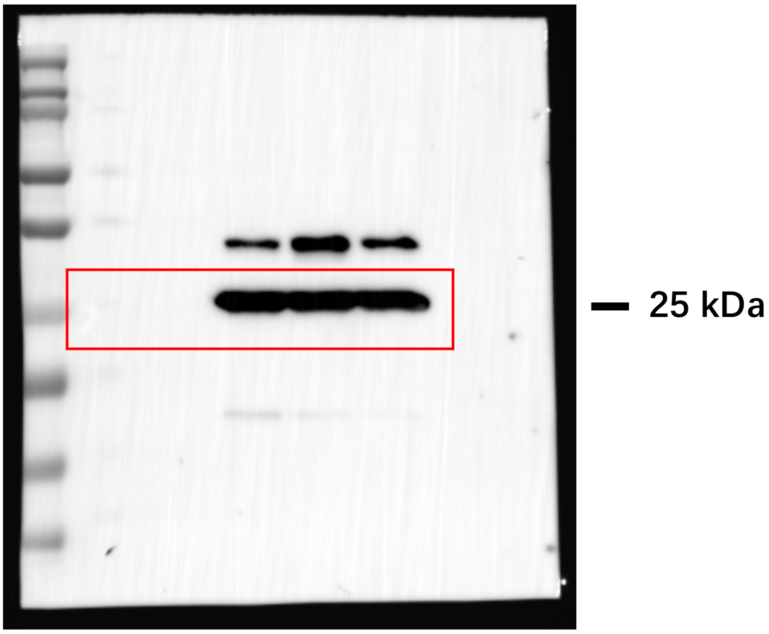

3、 **HA-IP**

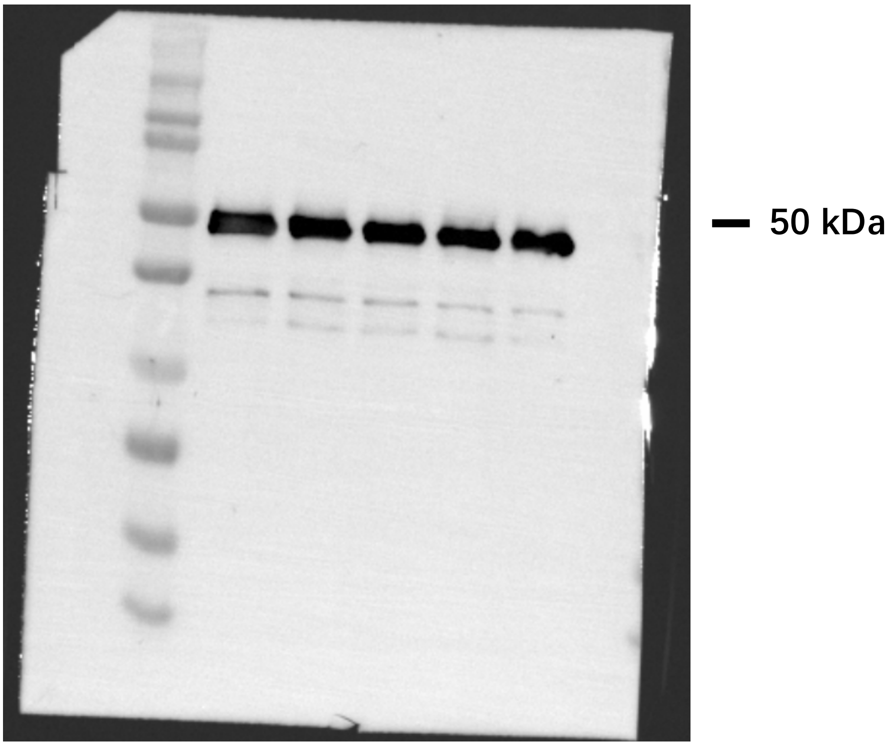

4、 **MYC-IP**

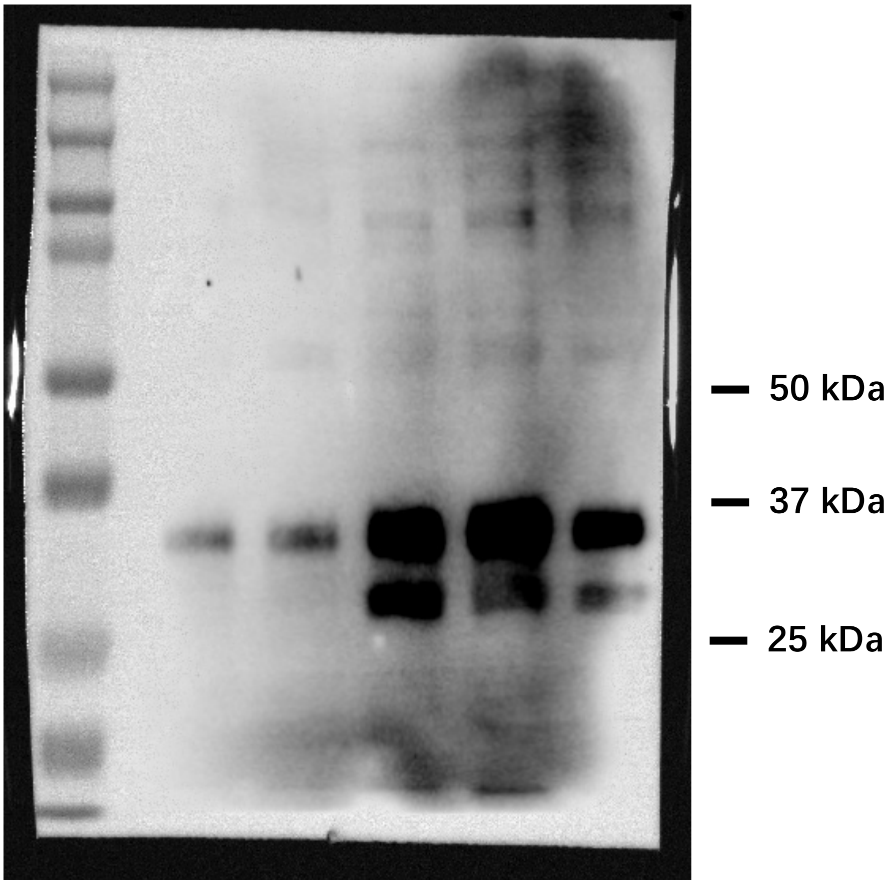

Supplement: Supplementary file 2 — Supporting Information [file ADVS-12-e04214-s001.pdf]
